# SWE-NEO: Swedish NEO-adjuvant trial comparing anti-PD-1 monotherapy to combined anti-CTLA-4/anti-PD-1 blockade in resectable stage III melanoma: study protocol for a phase III open-label multi-centre trial

**DOI:** 10.2340/1651-226X.2026.45174

**Published:** 2026-02-19

**Authors:** Hildur Helgadottir, Ana Carneiro, Francesca Portelli, Konstantinos Papadakis, Karl Björkström, Muyi Yang, Iva Johansson, Ingela Skogvall Svensson, Allan Jazrawi, Amanda Hallgren, Katja Harbst, Rusana Bark, Suzanne Egyhazi Brage, Anders Berglund, Karolin Isaksson, Stina Wickström, Jonas Nilsson, Lars Ny, Göran Jönsson, Roger Olofsson Bagge, the SWE-NEO Study Group

**Affiliations:** a Department of Oncology and Pathology, Karolinska Institutet, Stockholm, Sweden; b Theme Cancer, Karolinska University Hospital, Stockholm, Sweden; c Division of Oncology, Lund University Cancer Center, Department of Clinical Sciences, Lunds University, Lund, Sweden; d Oncology Unit, Department of Hematology, Oncology and Radiation Physics, Skåne University Hospital Comprehensive Cancer Center, Skåne University Hospital, Lund, Sweden; e Department of Clinical Pathology and Cancer Diagnostics, Karolinska University Hospital, Stockholm, Sweden; f Department of Clinical Pathology, Sahlgrenska University Hospital, Gothenburg, Sweden; g Institute of Biomedicine, Sahlgrenska Academy, University of Gothenburg, Gothenburg, Sweden; h Department of Clinical Genetics, Pathology and Molecular Diagnostics, Skåne University Hospital, Region Skåne, Lund, Sweden; i Department of Plastic surgery and Craniofacial surgery, Karolinska University Hospital, Stockholm Sweden; j Center for Clinical Cancer Studies, Karolinska University Hospital, Stockholm Sweden; k Division of Oncology, Department of Clinical Science, Faculty of Medicine, Lund University, Lund, Sweden; l Department of Clinical Sciences Intervention and Technology, Division of ENT Diseases, Karolinska Institutet, Stockholm, Sweden; m Epistat AB, Uppsala, Sweden; n Department of Clinical Sciences, Surgery, Lund University, Lund, Sweden; o Department of Surgery, Skåne University Hospital Kristianstad, Kristianstad, Sweden; p Department of Surgery, Institute of Clinical Sciences, Sahlgrenska Academy, University of Gothenburg, Gothenburg, Sweden; q Department of Oncology, Institute of Clinical Sciences, Sahlgrenska Academy at University of Gothenburg, Sahlgrenska University Hospital, Gothenburg, Sweden; r Department of Oncology, Sahlgrenska University Hospital, Gothenburg, Sweden; s Department of Surgery, Sahlgrenska University Hospital, Gothenburg, Region Västra Götaland, Sweden; t Wallenberg Centre for Molecular and Translational Medicine, University of Gothenburg, Gothenburg, Sweden

**Keywords:** melanoma, neoadjuvant therapy, immune checkpoint inhibitors, ipilimumab, nivolumab, PD-1, CTLA-4

## Background

In patients with stage III cutaneous melanoma, adjuvant treatments with PD-1 and BRAF+MEK inhibitors were approved based on improved recurrence-free survival (RFS) [1–3]. None of the adjuvant trials have yet demonstrated any benefit in overall survival (OS) while the OS data from the European Organisation for Research and Treatment of Cancer (EORTC) 1325/KEYNOTE-054 study are still awaited [4–6]. A population-based registry study from Sweden reported no significant differences in OS after the national implementation of adjuvant treatments in 2018 [7]. At the European Society for Medical Oncology (ESMO) meeting in September 2022, the results from the phase II SWOG S1801 study were presented, demonstrating significantly improved event-free survival (EFS) with neoadjuvant and adjuvant PD-1 inhibitors compared to only adjuvant anti-PD-1 [8]. Subsequently, already in October 2022, neoadjuvant treatment with PD-1 inhibitor monotherapy was implemented on a national level in Sweden [9]. The phase II PRADO (Personalized Response-driven Adjuvant combination therapy) trial further demonstrated the feasibility and clinical utility of a response-adapted approach in which pathologic response to neoadjuvant immune checkpoint blockade guided subsequent adjuvant therapy and surgical management. This strategy substantially reduced the need for therapeutic lymph node dissection (TLND) and adjuvant systemic treatment in patients achieving major pathologic response (MPR) [10]. Furthermore, at the American Society of Clinical Oncology (ASCO) meeting in June 2024, the results from the phase III NADINA study were presented, demonstrating improved EFS in patients receiving anti-PD-1 and anti-CTLA-4 combination as neoadjuvant treatment, with subsequent adjuvant therapy tailored according to pathologic response [11]. At present there are hence two studies demonstrating superiority with neoadjuvant compared to adjuvant treatment only, but no clinical trial has compared neoadjuvant PD-1 inhibitor monotherapy to PD-1 and CTLA-4 inhibitor combination therapy.

## Methods

### Aim

Two studies (SWOG S1801 and NADINA) have demonstrated superiority when using neoadjuvant treatment compared to adjuvant treatment only, but no study has compared anti-PD-1 monotherapy (SWOG 1801 regimen) to anti-PD-1/anti-CTLA-4 combination (NADINA regimen) therapy. The SWE-NEO study is a phase III randomised controlled multi-centre open-label trial, that aims to compare these two regimens in a response-guided approach, where the PD-1/CTLA-4 inhibitor combination is potentially more effective but also associated with more side effects.

### Study population

The study population includes patients with resectable stage III cutaneous, acral or unknown primary melanoma who are candidates for the neoadjuvant treatment procedure. Patients must be naïve to CTLA-4/PD-1/PD-L1 blockade and BRAF+MEK inhibition, and at least 18 years of age.

### Inclusion criteria

1. Age ≥ 18.
2. Signed and dated written informed consent.
3. Eastern Cooperative Oncology Group (ECOG) performance status of 0–1.
4. Patients must meet all the following criteria:
  - Histologically or cytologically confirmed stage III melanoma. In the case of in-transit metastases (with or without lymph node metastases)‚ ≤ 3 resectable in-transit metastases are permitted.
  - Patients with cutaneous, acral, or unknown primary melanomas are eligible for enrolment.
  - Resectable tumours are defined as having no significant vascular, neural, or bony involvement, and for which complete surgical resection with tumour-free margins can be safely achieved.
5. No history of other malignancies, except if treated with curative intent and with a cancer-related life expectancy of more than 5 years.
6. No prior therapy targeting CTLA-4, PD-1 or PD-L1.
7. No prior therapy targeting BRAF and/or MEK.

### Exclusion criteria

1. Unresectable melanoma.
2. Uveal/ocular or mucosal melanoma.
3. Any serious or uncontrolled medical conditions that, in the investigator’s opinion, may increase the risk associated with study participation or study drug administration, impair the subject’s ability to receive protocol therapy, or interfere with the interpretation of study results.
4. Any condition requiring systemic treatment with corticosteroids (>10 mg daily prednisone equivalents) or other immunosuppressive medications within 14 days prior of study drug administration.
5. Women who are pregnant or breastfeeding. Patients with childbearing potential must agree to safe contraception.
6. Any condition that may hamper compliance with the study protocol and follow-up schedule.

### Endpoints

The primary endpoint is EFS, defined as time from randomisation to melanoma progression (unresectable stage III or stage IV disease), melanoma recurrence, or death from any cause (treatment-related, melanoma related or any other).

The secondary endpoints of the SWE-NEO trial are:

1. RFS, defined as the time from the date of surgery to the date of melanoma recurrence, treatment-related death or melanoma-related death, whichever occurs first.
2. Distant metastasis-free survival (DMFS), defined as the time from the date of randomisation to the date of first distant metastasis, treatment-related death or melanoma-related death, whichever occurs first.
3. OS, defined as time from the date of randomisation to the date of death from any cause.
4. MPR, defined as ≤10% viable tumour cells in the excised tumour.
5. Correlation of pathologic response in each arm to RFS, DMFS, and OS.
6. Correlation of radiological and clinical response evaluation to RFS, DMFS, and OS.
7. Proportion of patients having surgery according to protocol-defined timing (within 10 weeks from first neoadjuvant treatment course).
8. Surgical complication rates graded according to Clavien-Dindo surgical classification.
9. Frequency and duration of all grade and grade 3–5 treatment- related adverse events (AEs), graded according to Common Terminology Criteria for Adverse Events (CTCAE) 5.0.

Explorative endpoints include different biological markers analysed from sequential blood and tumour samples and their correlation with treatment efficacy and safety. The following will be studied as potential biomarkers:

1. Circulating tumour DNA (ctDNA) in plasma.
2. Extracellular vesicles (EVs) in plasma.
3. Multiparameter flow cytometry, and to profile immune cells in peripheral blood mononuclear cells (PBMC) isolated from blood.
4. Single cell analyses of tumour and blood samples including single cell RNA sequencing and T cell and B cell receptor profiling to determine predictive biomarkers.
5. Tumour spatial transcriptomic analyses to determine tumour architectural features that associate with pathological response and EFS.

### Study intervention

**Figure 1:**
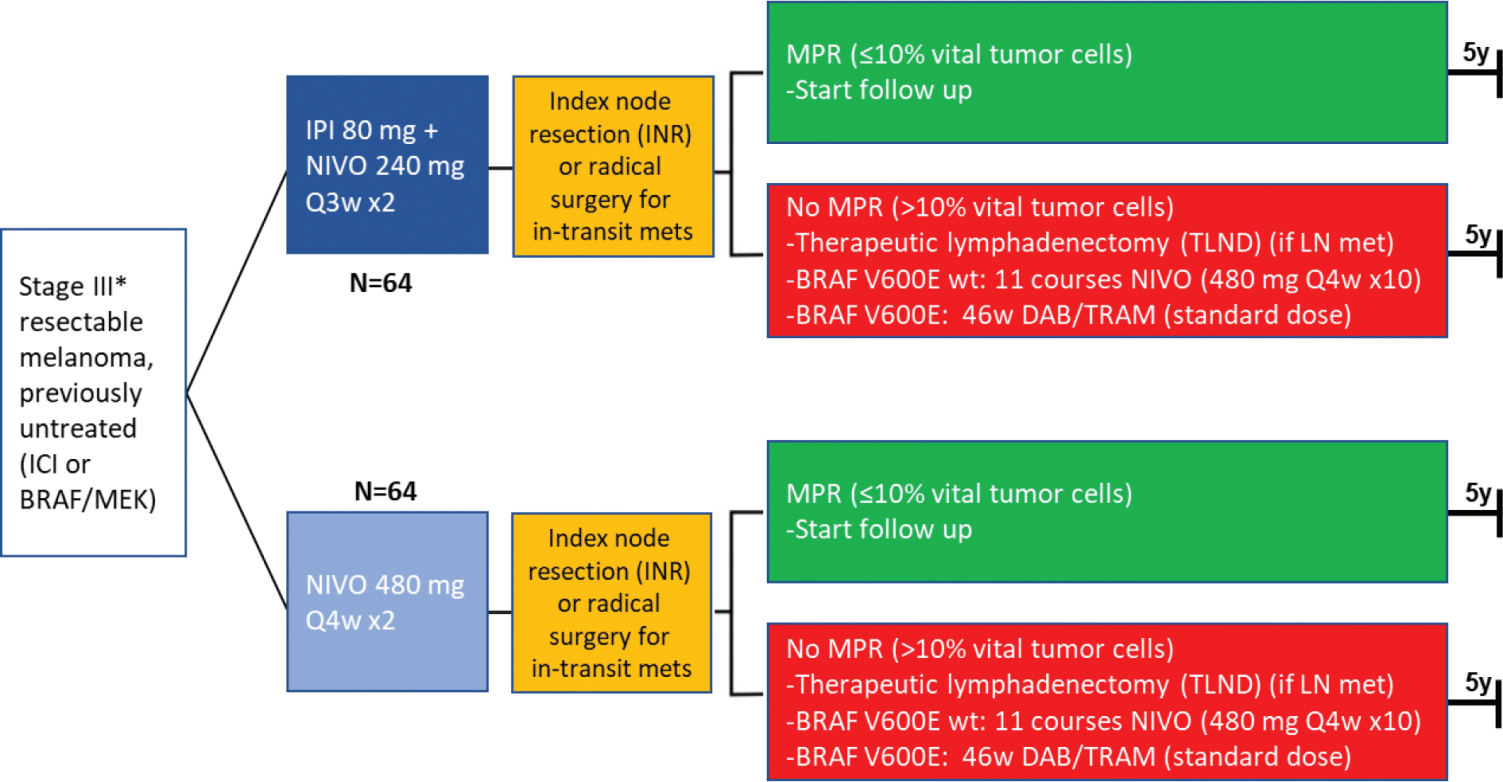
Trial overview. ICI: Immune checkpoint inhibitors. IPI: Ipilimumab, NIVO: Nivolumab, MPR: Major pathological response, LN: Lymph node. *Up to three resectable in-transit metastases are permitted, where included patients are stratified according to the presence or absence of in-transit metastases.

Patients will be randomised after a diagnosis of resectable stage III melanoma to receiving either two cycles of intravenous infusion with ipilimumab 80 mg plus nivolumab 240 mg (combination therapy administered at 3-weeks interval) or two cycles of intravenous infusion with nivolumab 480 mg (monotherapy administered at 4-weeks interval) (Figure 1). In both groups, the two neoadjuvant treatment courses will be followed by an index node resection (in patients with lymph node metastases) or radical surgery for in-transit metastases. Patients with MPR, receive no further treatment and will enter follow-up. In the absence of MPR, patients with lymph node metastases undergo TLND and then receive adjuvant treatment, nivolumab 480 mg (Q4w x12), or if harbouring BRAF V600E tumour mutation, 46 weeks of dabrafenib 150 mg x2 and trametinib 2 mg 1×1.

### Study design

The study is a phase III superiority trial comparing neoadjuvant anti-PD-1 monotherapy to anti-CTLA-4/anti-PD-1 combination therapy in patients with resectable stage III–IV melanoma with EFS as the primary endpoint. The sample size and power calculations are based on a two-sided type 1 error of 5% and a statistical power of 70%. A power of 70% was selected as a deliberate balance between the risk of a false-negative conclusion and the feasibility constraints imposed by a limited recruitable population and event accrual within a reasonable study timeframe. Included patients are stratified according to the presence or absence of in-transit metastasis. Randomisation is 1:1 with a total sample size of 128 subjects (64 per arm), with a total number of 85 events required to detect a difference in EFS of 13% or higher between the two groups. This is based on indirect cross-trial comparison of 24-month EFS rates between neoadjuvant pembrolizumab in SWOG S1801 (EFS 72% at 24 months) and neoadjuvant ipilimumab+nivolumab in NADINA (EFS 77% at 24 months). However, this cross-trial difference is expected to underestimate the true regimen effect due to differences in patient populations, perioperative systemic therapy exposure, and follow-up structures. On this basis, SWE-NEO prespecifies a clinically meaningful absolute EFS difference of 13%, representing a benefit magnitude that would justify the additional toxicity of the added CTLA-4 blockade and remains conservative relative to the ~20–25% absolute EFS gains observed when neoadjuvant strategies are compared with adjuvant-only approaches. Included patients are stratified for in-transit metastases. The anticipated accrual time is 2 years, and the follow-up period is 5 years.

### Ethical consideration

In resectable stage III melanoma, both neoadjuvant PD-1 inhibitor monotherapy and PD-1 and CTLA-4 inhibitor combination have been shown to result in improved EFS and DMFS compared to adjuvant-only treatment. As of December 2025, neither regimen has been approved by the EMA or the FDA. However, in Sweden, both these regimens have been approved for neoadjuvant treatment in resectable stage III melanoma. The PD-1/CTLA-4 inhibitor combination is potentially more effective, but also associated with increased toxicity and higher treatment costs. In patients presenting with resectable stage III melanoma, acceptance of the risk of serious, life-threatening, or chronic side effects is generally lower than in the setting of inoperable metastatic disease. Therefore, it is possible that PD-1 inhibitor monotherapy regimen is sufficient in this clinical setting. It is therefore essential to study this benefit/risk balance to determine which treatment strategy is most beneficial. In addition, translational analyses aim to identify biomarkers that may guide treatment selection for individual patients.

Patient representatives from the Swedish melanoma patient association (Melanomföreningen) were consulted during protocol development to ensure patient relevance and acceptability, and they expressed support for the final study design. The study will be conducted in accordance with Good Clinical Practice (GCP) guidelines and the Declaration of Helsinki. All participants will receive oral and written information regarding risks of participation and their right to withdraw from the study at any time. All participants are required to provide signed and dated written informed consent (available in the Supplementary Material) prior to participation. The trial protocol is reported in accordance with the SPIRIT guidelines for clinical trials (see Supplementary Material). The trial was approved on April 30th, 2025, by the EMA Clinical Trials Information System (CTIS) (trial ID: 2024-519593-39-00) and by the Swedish Biobank Authority. ClinicalTrials.gov identifier is NCT06794775, registered on January 22nd, 2025.

## Perspectives

SWE-NEO is a fully academic trial comparing two courses of either nivolumab monotherapy or nivolumab plus ipilimumab in the neoadjuvant setting. The feasibility of fully recruiting the trial within 2 years is based on the national experience with neoadjuvant treatment since its implementation in Sweden as clinical routine in 2022 [9]. The response adapted switch to BRAF/MEK inhibition in both arms will aid to give insights on the true effect on outcome comparing neoadjuvant combination or monotherapy, where such adaptive approach was only applied in NADINA and not in SWOG S1801, which obscures the comparison between these two studies. The results are expected to provide valuable insights to define the optimal neoadjuvant approach for resectable stage III melanoma. The results of the trial are expected to directly inform clinical decision-making by identifying the regimen that provides the best balance of efficacy and safety, potentially shaping future standard of care.

## Trial status

As of December 2025, the SWE-NEO trial is open at the three participating sites, in Stockholm (Karolinska University Hospital), Gothenburg (Sahlgrenska University Hospital) and Lund (Skåne University Hospital) and all sites have initiated patient enrolment. Further details on the SWE-NEO trial are provided in the study protocol in the Supplementary Material.

## Supplementary Material







## Data Availability

Not applicable, no data are presented, as this is a report of a study protocol.
