# Supplementary material for: SWE-NEO: Swedish NEO-adjuvant trial comparing anti-PD-1 monotherapy to combined anti-CTLA-4/anti-PD-1 blockade in resectable stage III melanoma: study protocol for a phase III open-label multi-centre trial
Source: Acta Oncol. 2026 Feb 19;65:45174. doi: 10.2340/1651-226X.2026.45174 (PMC12930509; doi:10.2340/1651-226X.2026.45174)
Supplement: Supplementary file 2 [file AO-65-45174-s2.pdf]

Supplementary material has been published as submitted. It has not been copyedited, or typeset by Acta Oncologica

SWE-NEO: Svensk studie som jämför neoadjuvant anti-PD-1 monoterapi med kombinerad anti-CTLA-4/anti-PD-1 terapi för patienter med operabelt stadium III-melanom

SWE-NEO-studien

Information till försökspersonerna

Du tillfrågas härmed om du vill delta i en klinisk studie som genomförs vid Karolinska universitetssjukhuset, Sahlgrenska universitetssjukhuset och Skånes universitetssjukhus, där vi vill jämföra två typer av immunterapi som ges före din planerade operation för melanom, det vill säga neoadjuvant behandling. Denna information beskriver varför studien görs, hur den går till, tänkbara risker och obehag, samt hur insamlade uppgifter kommer att behandlas. Ta god tid på dig att läsa informationen, och fråga gärna om något är oklart. Studien är godkänd av Etikprövningsmyndigheten och Läkemedelsverket. Karolinska universitets-sjukhuset är sponsor (ansvarig organisation) för studien. Totalt kommer 128 patienter att inkluderas i denna kliniska studie.

Bakgrund och syfte

Melanom som uppstår i huden behandlas i första hand med operation, vilket oftast är en botande behandling. För patienter där melanomet spridit sig till närliggande hud eller lymfkörtlar kan det bli aktuellt även med medicinsk onkologisk behandling med immunterapi. Hos patienter med operabla lymfkörtel- eller hudmetastaser så rekommenderas numera att man först ger behandling med immunterapi och sedan opererar bort metastaserna, det som kallas för neoadjuvant immunterapibehandling. Syftet med immunterapin är att behandla all eventuell spridning i kroppen och därmed på sikt minska risk för återfall och därmed död i sjukdomen. Baserat på två tidigare studier, som båda visat positiva resultat, så finns det i dagsläget två olika godkända behandlingsupplägg i Sverige, den ena behandling med ett preparat (singelbehandling), en så kallad PD-1-hämmare, den andra med en kombination (kombinationsbehandling) av två olika preparat med PD-1 hämmare och CTLA-4 hämmare, före den planerade operationen. Dessa olika upplägg har hittills inte jämförts med varandra, och det är det som görs i denna studie. Kombinationsbehandlingen kan potentiellt ge bättre effekt på tumören, men det är även känt att den ger mer biverkningar. Det är därför viktigt att utvärdera dessa behandlingsupplägg sida vid sida för att veta vad som gagnar patienterna mest, både när man ser till effekt på tumören och biverkningar.

Hur går studien till?

Bedömning för att kunna ingå i studien
Studien börjar med ett screeningbesök där du undersöks för att se om studien är lämplig för dig. Det som gjort att du kan komma att inkluderas i denna studie är att man sett att det hos dig finns lymfkörtel- och/eller hudmetastaser av melanom som bedöms kunna opereras bort. Enligt nuvarande rutin så rekommenderas då neoadjuvant immunterapibehandling innan operationen. Du kommer bli tillfrågad att delta i studien i vilken man lottas till singel immunterapibehandling med nivolumab (PD-1-hämmare) eller kombinationsbehandling med ipilimumab och nivolumab (CTLA-4 och PD-1 hämmare). Bestämmer du dig för att vara med i studien undertecknar du först samtyckesformuläret. Din läkare gör en bedömning av ditt allmäntillstånd och dina tumörer och du kommer att få lämna rutinblodprover som är samma som de man brukar ta inför immunterapibehandling. Alla kvinnor i fertil ålder får lämna ett graviditetstest (blodprov) för att utesluta graviditet Det kommer även att tas blodprov och tumörbiopsi för forskningsändamål, se avsnitt nedan om provtagning. Din senaste röntgenundersökning, DT (datortomografi) eller PET-DT (positronemissionstomografi-DT) kommer användas som baslinjeundersökning om du går med i studien. Om undersökningen är för gammal eller ofullständig kan man behöva göra en ny baslinjeundersökning. Du får även lämna ett EKG för att undersöka din hjärtfunktion, vilket även är rutin för immunterapi-behandling. När undersökningarna är klara och man ser att du fortsatt är lämplig för studien, så kommer du lottas till singel- eller kombinationsbehandling.

Neoadjuvant behandling
Lottas du till singelbehandlingen så får du två kurer nivolumab med 4 veckors mellanrum och därefter planeras operationen. Lottas du till kombinationsbehandlingen får du två kurer med ipilimumab och nivolumab, med 3 veckors mellanrum och därefter planeras operationen. Oavsett behandling så ges den som dropp enligt klinisk rutin för dessa läkemedel. Förväntade biverkningar av immunterapin är immunrelaterade med inflammation som kan uppstå i olika organ eller vävnader, se även avsnitt nedan om möjliga risker med att delta i studien. Din läkare kommer ge dig mer information om förväntade biverkningar av immunterapin. Om du får några svårare biverkningar av den första immunterapikuren så kan det bedömas som för riskfyllt att ge den andra kuren. Då planeras istället operationen in redan efter en kur. Efter att du fått den neoadjuvanta behandlingen så görs en ny röntgenundersökning som jämförs med den du gjorde innan behandlingsstart. Efter det görs det en bedömning av röntgenundersökningen, blodproverna, ditt allmäntillstånd samt om det uppkommit några biverkningar eller andra hälsorelaterade effekter, detta för att se att det inte finns något som förhindrar att man går vidare med den planerade operationen.

Operation

Efter den neoadjuvanta behandlingen planeras en operation för att ta bort metastaser. För att man vid operationen lätt ska kunna identifiera tumören (som kan ha minskat av den neoadjuvanta behandlingen), så kommer de flesta patienter att behöva göra en så kallad markering av tumören, även detta är i enlighet med vanlig klinisk rutin. Behovet av en sådan markering beror på typen och utseendet av din metastas. Markeringen görs innan den neoadjuvanta behandlingen, under vägledning med ultraljud sticks en liten nål in i tumören och en markör placeras. Operationens upplägg ser olika ut beroende på typen och omfattning av tumören och var på kroppen den sitter. Din läkare kommer ge dig mer information om hur din operation kommer att gå till och vad som kan förväntas under och efter operationen, samt risker med operation och narkos. Det är vanligt att man blir inneliggande ett fåtal dagar och att man får ett drän som sitter kvar under en viss tid efter operationen. Se även avsnitt nedan om möjliga risker med att delta i studien.

Adjuvant behandling och uppföljning

Efter operationen gör patologen en undersökning och bedömning av din bortopererade tumör. Man undersöker om det finns kvarvarande levande tumörceller i metastasen. Hos en andel av patienterna vet man att den neoadjuvanta behandlingen gör att det inte finns några, eller nästan inga levande tumörceller kvar. Om detta är vad man ser i din tumör så är du färdigbehandlad och du kommer inleda uppföljning med röntgenundersökningar och kliniska kontroller. Om patologen ser kvarvarande levande tumörceller i din bortopererade tumör, så kommer du få mer behandling. Hos många, men inte alla (åter beroende på typ och läge av tumören) görs det en full lymfkörtelutrymning på stället där du tidigare opererats. Din läkare kommer ge dig mer information om hur denna operation kommer att genomföras och vad som kan förväntas under och efter operationen. Därefter ges en medicinsk efterbehandling som planeras pågå i ca 10 månader, och kallas för adjuvant behandling (efterbehandling). Om man i din tumör sett att det finns en särskild mutation i en gen som heter BRAF, så kommer din adjuvanta behandling bestå av tabletter som du tar dagligen av ett läkemedel som kallas för BRAF- och MEK-hämmare (dabrafenib och trametinib). Om det däremot inte finns någon sådan mutation, så kommer din adjuvanta behandling att bestå av immunterapi med nivolumab, som ges som dropp var fjärde vecka. Adjuvant immunterapi ges förutsatt att det inte uppkommit några allvarliga immunrelaterade biverkningar under den neoadjuvanta behandlingen, eller annat som rör din hälsa uppkommit som gör att man bedömer att det är för riskfyllt att ge mer behandling, och du startar då i stället din uppföljning. Den adjuvanta behandlingen ges enligt det som är klinisk rutin och din läkare kommer ge dig mer information om behandlingen och förväntade biverkningar. Om du skulle få ett nytt återfall eller oacceptabla biverkningar så avbryter man den adjuvanta behandlingen tidigare än de tilltänkta 10 månaderna och du startar då istället din uppföljning. Förfarandet efter operation med olika åtgärder beroende på behandlingssvar och tumörmutation är också enligt det som

rekommenderas i klinisk rutin. Oavsett adjuvant behandling så kommer du att gå på röntgenundersökningar och kliniska kontroller i totalt 3 år efter senaste operationen, vid cirka 3, 6, 9, 12, 18, 24, 30 och 36 månader.

Vilka andra behandlingar finns det?

Om du inte vill delta i denna studie för behandling av malignt melanom, kan vi förklara andra behandlingsalternativ som finns för dig.

Provtagning

Blodproverna som tas för forskningsändamål under studien sker vid maximalt 4 tillfällen med 130 ml per provtagning och uppgår till en maximal totalvolym på 520 ml (som referens så är den blodvolym som lämnas vid en vanlig blodgivning ca 450 ml). Innan start av den neoadjuvanta behandlingen så tas det även ett prov (biopsi) för forskning från din tumör. Detta görs genom att sticka en nål i tumören, där man vid samma tillfälle gör två dylika provtagningsstick i din tumör. Vid din operation kommer även en del av din tumör samlas in för forskning. Skulle tumören tillväxa eller om du skulle få ett återfall med nya tumörer så kommer det även tas prov från dessa för forskning, antingen med två mellannålsbiopsier eller genom operation av tumören. Syftet med studiens provtagning av blod och tumör är att undersöka olika biologiska faktorers inverkan på hur du svarar på behandlingen. Proverna kommer att analyseras på forskningslaboratorium på Karolinska Institutet, Göteborgs universitet och Lunds universitet.

Biobank

Alla prover (blod och tumörvävnad) som tas för forskningsändamål i studien kommer att tillhöra en så kallad biobank: Stockholms Medicinska Biobank (nr 914) i enlighet med Biobankslagen (2023:38). Proverna kommer att vara märkta enligt sjukhusets ordinarie rutiner och kommer endast att användas för de ändamål som angivits ovan. De kan endast bli aktuella för fler analyser, om du lämnat ett nytt samtycke, och/eller nytt godkännande erhållits av Etikprövningsmyndigheten. Du har rätt att utan förklaring begära att dina prover skall förstöras. Alla prover kommer att förvaras kodade vilket innebär att de endast kan härledas till dig som person med hjälp av en ”kodnyckel” som beskrivs i avsnittet ”Vad händer med mina uppgifter?” nedan. Dina prover kommer att sparas i 20 år efter avslutad studie. Både blod- och tumörprover som skickas för analys förbrukas helt vid analysen, men de prover som finns kvar i biobanken sparas (i 20 år). Ditt samtycke till att prover sparas i en biobank är frivilligt. Du kan när som helst dra tillbaka ditt samtycke och utan förklaring begära att dina sparade prover förstörs. Den information som har samlats in fram till dess att du väljer att avbryta studien kommer dock att finnas kvar.

Restriktioner (vad förväntas av dig?)

Det är viktigt att du kommer på alla schemalagda besök och följer alla procedurer i studien. Det är också viktigt att du talar om för oss det som rör din hälsa, före och under studien.

Möjliga risker med att delta i studien

Behandlingarna som vi undersöker i denna studie är etablerade behandlingar för ditt tillstånd (melanom med operabla metastaser). Det innebär att i klinisk rutin så skulle du rekommenderas likartad behandling som den som ges i studien. I studien jämförs två olika godkända behandlingar där det i nuläget inte finns kunskap om vilken som är mer fördelaktig. Risker med immunterapi, både den som ges neoadjuvant och som vissa även får adjuvant (efter operation) är inflammationer (autoimmuna reaktioner) som kan uppstå i olika vävnader och t.ex. ge symtom i mag-tarmkanalen (diarré, illamående, magbesvär), hudreaktioner (utslag och klåda), hormonstörningar (hyper-/hypotyreos och hypofysit), smärta i leder/muskler och svullna leder, eller symtom från andningsorganen (andnöd eller hosta). De typer av biverkningar som uppstår vid singelbehandling med nivolumab eller kombinationsbehandling med ipilimumab och nivolumab är av samma slag, men av en högre frekvens och allvarlighetsgrad med kombinationsbehandlingen. De besök som görs i studien skiljer sig inte från vanlig behandlingsrutin och innebär, förutom studiespecifik provtagning som beskrivs ovan, inga extra besök eller undersökningar för dig. Vid provtagning (både av blod och tumör) finns alltid en liten risk för blödning eller obehag vid stickstället, samt en infektionsrisk. Uppföljning med radiologi (skiktröntgen) sker mer frekvent och under längre tid än vad som är klinisk rutin i Sverige (upp till sex fler undersökningar tillkommer), som dock inte är fler radiologiska undersökningar än vad som internationellt betraktas som klinisk rutin för detta tillstånd. När en skiktröntgen genomförs så utsätts du för röntgenstrålning som ger en mycket liten ökad risk för att drabbas av annan cancer. Vid operation finns det risker då det kan uppstå t.ex. blödningar och infektioner eller komplikationer relaterade till narkosen, men även detta är i enlighet med vanlig klinisk rutin.

Finns det några fördelar med att delta?

Vår förhoppning är att studiebehandlingen kommer att hjälpa dig, men detta kan inte garanteras. Många upplever dock den täta kontakten med läkare och sjuksköterska som något positivt. Resultaten från studien kan komma att hjälpa oss att behandla patienter med malignt melanom mer framgångsrikt i framtiden.

Vad händer med mina uppgifter?

Alla uppgifter som samlas in om dig i studien kommer att hanteras enligt EU:s dataskyddsförordning (General Data Protection Regulation, GDPR) och alla persondata

kommer att hanteras i kodad form. Studieresultaten kommer att hanteras i kodad form och endast personer ansvariga för studien kommer att ha tillgång till den kodnyckel som kopplar resultaten till respektive studieperson. Ansvarig för dina personuppgifter är [Karolinska Universitetssjukhuset ANPASSA FÖR VARJE STUDIESITE] Enligt dataskyddsförordningen har du rätt att kostnadsfritt få ta del av de uppgifter om dig som hanteras i studien, och vid behov få eventuella fel rättade. Om du vill ta del av uppgifterna ska du kontakta studieansvarig läkare Hildur Helgadottir (se kontaktuppgifter nedan). Dataskyddsombud nås på [dataskyddsombud. karolinska@regionstockholm.se, Tel: 08-517 700 00 ANPASSA FÖR VARJE STUDIESITE] Om du är missnöjd med hur dina personuppgifter behandlas har du rätt att ge in klagomål till Integritetsskyddsmyndigheten, som är tillsynsmyndighet. Om Du vill dra tillbaka ditt samtycke, begränsa användningen av data som samlats in om dig, kontakta studieansvarig läkare (se nedan).

Hur får jag information om resultatet av studien?

När studien är avslutad kommer data att analyseras och presenteras i vetenskapliga tidskrifter. Data presenteras alltid på ett sådant sätt att det inte går att spåra tillbaka till dig som individ. Önskar du få del av data kontaktar du studieansvarig läkare. Du har ingen skyldighet att ta del av analysresultat om du inte önskar.

Försäkring och ersättning

Du är fullt försäkrad som deltagare i denna studie. Eventuella skador som uppkommer i samband med den vård du får i studien täcks av patientskadeförsäkringen och skador som kan ha orsakats av det studieläkemedel du får täcks av läkemedelsförsäkringen. Det utgår ingen ersättning för studiedeltagande, men deltagandet innebär heller inga extra kostnader för dig. Ersättning för resor betalas inte ut.

Deltagandet är frivilligt

Ditt deltagande är frivilligt och du kan när som helst välja att avbryta deltagandet. Om du väljer att inte längre delta eller vill avbryta ditt deltagande behöver du inte uppge varför, och det kommer inte heller att påverka din framtida vård eller behandling. Data som redan insamlats kommer att behållas.

Ansvarig för studien
Hildur Helgadottir, överläkare, Tema cancer, Karolinska Universitetssjukhuset
tfn 08-123 73415, hildur.helgadottir@regionstockholm.se
Forskningssköterska: *Centerspecifika kontaktuppgifter läggs in innan studiestart*

Samtycke till studiedeltagande i SWE-NEO-studien

- Jag har erhållit muntlig och skriftlig information om studien, jag har fått möjlighet att ställa frågor och fått tid att fundera på mitt beslut och jag samtycker till deltagande.
- Jag har fått information om att denna studie är granskad och godkänd av Etikprövningsmyndigheten och Läkemedelsverket.
- Jag är medveten om att medverkan i studien är helt frivilligt och jag kan när som helst avbryta mitt deltagande utan att motivera varför. Kvaliteten på min vård kommer inte att påverkas av detta.
- Jag samtycker till att mina personuppgifter används i enlighet med Dataskydds förordningen (GDPR) i samband med denna forskning och att personer som arbetar med forskningsprojektet tar del av min journal. Efter skriftlig begäran kan jag utan kostnad få ett utdrag med de personuppgifter som registrerats en gång per år, står något felaktigt skall detta korrigeras.
- Jag samtycker till att mina prover sparas i en biobank på det sätt som beskrivs i försökspersonsinformationen.

________________________________	________________________________
Underskrift försöksperson	Namn (textat)
________________________________	________________________________
Personnummer	Datum
________________________________	________________________________
Underskrift ansvarig läkare	Namn (textat)
	________________________________
	Datum
