# Supplementary material for: SWE-NEO: Swedish NEO-adjuvant trial comparing anti-PD-1 monotherapy to combined anti-CTLA-4/anti-PD-1 blockade in resectable stage III melanoma: study protocol for a phase III open-label multi-centre trial
Source: Acta Oncol. 2026 Feb 19;65:45174. doi: 10.2340/1651-226X.2026.45174 (PMC12930509; doi:10.2340/1651-226X.2026.45174)
Supplement: Supplementary file 3 [file AO-65-45174-s3.pdf]

Supplementary material has been published as submitted. It has not been copyedited, or typeset by Acta Oncologica

Trial ID:	SWE-NEO Trial
Version No:	2.0
Date:	2025-04-07
EU Trial Number:	2024-519593-39-00

CLINICAL TRIAL PROTOCOL

SWE-NEO: Swedish NeoAdjuvant Trial Comparing anti-PD-1 Monotherapy to Combined anti-CTLA-4/anti-PD-1 blockade in Resectable Stage III Melanoma

Trial ID:	SWE-NEO Trial
EU Trial number:	2024-519593-39-00
Version number:	2.0
Date:	2025-04-07
Sponsor:	Karolinska University Hospital, Sweden
Sponsor representative:	Hildur Helgadottir

Table Of Contents

Revision history 5
Signature page 6
Contact information 7
List of used acronyms and abbreviations 9
1. Synopsis 12
2. Background and rationale 16
3. Benefit-risk evaluation 20
4. Trial objectives 21
4.1. Primary objective 21
4.2. Secondary objective(s) 21
4.3. Exploratory objectives 22
4.4. Primary endpoint 22
4.5. Secondary endpoints 22
4.6. Exploratory endpoints 22
5. Trial design and procedures 23
5.1. Overall trial design 23
5.2. Procedures and flow chart 24
5.3. Biological sampling procedures 29
5.3.1. Handling, storage, and destruction of biological samples 29
5.3.2. Total volume of blood per subject 29
5.3.3. Biobank 30
5.4. Start, end, temporary halt and early termination 31
5.4.1. Start of the clinical trial 31
5.4.2. Temporary halt or early termination 31
5.4.3. End of the clinical trial 31
6. Subject selection 31
6.1. Inclusion criteria 31
6.2. Exclusion criteria 33
6.3. Screening and inclusion 33
6.4. Withdrawal criteria 34
7. Trial treatments 34
7.1. Description of investigational medicinal product(s) 35

7.1.1. Dose and administration ... 36
7.1.2. Packaging, labelling, and handling of investigational medicinal products(s) ... 36
7.1.3. Drug accountability and treatment compliance ... 36
7.1.4. Randomisation ... 36
7.1.5. Blinding ... 36
7.1.6. Destruction ... 37
7.2. Auxiliary medicinal products ... 37
7.2.1. Dose and administration ... 37
7.2.2. Drug accountability and treatment compliance ... 37
7.3. Concomitant use of other medicinal products and treatments ... 38
7.4. Treatment after trial end ... 38
8. Methods for measurement of endpoints for clinical efficacy and safety ... 38
8.1. Methods for measurement of endpoints for clinical efficacy ... 38
8.1.1. Primary endpoint ... 38
8.1.2. Secondary endpoints ... 38
8.1.3. Exploratory endpoints ... 41
8.2. Methods for measurement of endpoints for clinical safety ... 41
9. Handling of Adverse Events ... 42
9.1. Definitions ... 42
9.1.1. Adverse Event (AE) ... 42
9.1.2. Adverse Reaction (AR) ... 43
9.1.3. Serious Adverse Event (SAE) ... 43
9.1.4. Suspected Unexpected Serious Adverse Reaction (SUSAR) ... 43
9.2. Assessment of Adverse Events (AE) ... 43
9.2.1. Assessment of causal relationship ... 43
9.2.2. Assessment of intensity ... 44
9.2.3. Assessment of seriousness ... 45
9.3. Reporting and registration of Adverse Events ... 45
9.3.1. Reporting of Adverse Events (AE) ... 46
9.3.2. Reporting of Serious Adverse Events (SAE) ... 46
9.3.3. Reporting of Suspected Unexpected Serious Adverse Reactions (SUSAR) ... 47
9.4. Follow-up of Adverse Events ... 47
9.5. Independent Data Monitoring Committee ... 47
9.6. Annual Safety Report (ASR) ... 48

9.7. Procedures in case of emergencies, overdose or pregnancy 48
10. Statistics 49
10.1. Analysis population 50
10.2. Statistical analyses 50
10.2.1. Statistical methods 50
10.2.2. Drop-outs 50
10.3. Adjustment of significance and confidence interval 50
10.4. Sample size calculations 51
10.5. Interim analysis (if relevant) 51
11. Quality Control and Quality Assurance 51
11.1. Quality Assurance and Sponsor oversight 52
11.2. Monitoring 52
11.3. Source data 53
11.4. Deviations, serious breaches and other reporting obligations 53
11.5. Audits and inspections 54
12. Ethics 54
12.1. Compliance to the protocol, ICH-GCP and regulations 54
12.2. Ethical review of the trial 55
12.3. Procedure for obtaining informed consent 55
12.4. Data protection 56
12.5. Insurances 57
13. Substantial changes to the trial 57
14. Collection, handling, and archiving of data 57
14.1. Case Report Form 58
15. Notification of trial completion, reporting, and publication 58
16. References 60

Revision history

Protocol version	Date of Issue	Summary of changes<br><br>*Describe all changes since the first final protocol.*
2.0	2025-04-07	Exclusion criteria no 3 clarified<br><br>Biological sampling procedures section 5.3.1 modified

Signature page

Sponsor

I am responsible for ensuring that this protocol includes all essential information to be able to conduct this trial. I will submit the protocol and all other important trial-related information to the responsible investigator(s) so that they can conduct the trial correctly. I am aware that it is my responsibility to hold the staff members who work with this trial informed and trained.

____

Signature of sponsor representative Date

Hildur Helgadottir

Principal Investigator

I have read this protocol and agree that it includes all essential information to be able to conduct the trial. By signing my name below, I agree to conduct the trial in compliance with this clinical trial protocol, the EU Regulation on clinical trials of medicinal products for human use (EU 536/2014), the Declaration of Helsinki, ICH-GCP (Good Clinical Practice) guidelines and the current national regulations governing the conduct of this clinical trial.

I will submit this protocol and all other important trial-related information to the staff members and investigators who participate in this trial, so that they can conduct the trial correctly. I am aware of my responsibility to continuously keep the staff members and investigators who work with this trial informed and trained.

I am aware that quality control of this trial will be performed in the form of monitoring and eventual audit and inspection.

____

Principal Investigator’s signature Date

____

Printed name

Contact information

**Responsibility in the clinical trial**	
Sponsor representative/ Coordinating Investigator	Hildur Helgadottir, MD, PhD<br>Karolinska Comprehensive Cancer Center<br>Karolinska University Hospital<br>171 76 Stockholm<br>+46 (0)707 557722<br>hildur.helgadottir@regionstockholm.se
Principal Investigators	*Coordinating investigator, principal investigator Stockholm:*<br>Hildur Helgadottir, Associate professor, Oncologist, Karolinska Comprehensive Cancer Center<br>hildur.helgadottir@regionstockholm.se<br><br>*Principal investigator Gothenburg, Oncology Unit:*<br>Lars Ny, professor, Oncologist,<br>Sahlgrenska Comprehensive Cancer Center<br>lars.ny@vgregion.se<br><br>*Sub-investigator Gothenburg, Leading surgeon for SWE-NEO:*<br>Roger Olofsson Bagge, Professor, Surgeon,<br>Sahlgrenska Comprehensive Cancer Center<br>roger.olofsson@gu.se<br><br>*Principal investigator Skåne:*<br>Anna Carneiro, Associate professor, Oncologist, Skåne University Hospital Comprehensive Cancer Center<br>Ana.SequeiraDeVasconcelosDiasCarneiro@skane.se
Responsible for translational analyses of biological study samples	Göran Jönsson, Professor,<br>Lund University<br>goran_b.jonsson@med.lu.se<br><br>Stina Wickström, Associate professor<br>Karolinska Institutet<br>stina.wickstrom@ki.se<br><br>Jonas Nilsson, Professor, University of Gothenburg<br>jonas.a.nilsson@surgery.gu.se
Other study responsibilities	Pathology representative Stockholm:<br>Francesca Portelli<br>francesca.portelli@regionstockholm.se<br><br>Surgery representatives Stockholm:

	Alberto Falk Delgado<br>alberto.falk-delgado@regionstockholm.se<br>Allan Jazrawi<br>allan.jazrawi@regionstockholm.se<br><br>Radiology representative Stockholm:<br>Lennart Blomqvist<br>lennart.k.blomqvist@regionstockholm.se<br><br>Pathology representative Gothenburg:<br>Iva Johansson<br>Iva.johansson@vgregion.se<br><br>Radiology representative Gothenburg:<br>Andrew Wong<br>wing-kee.wong@vgregion.se<br><br>Surgery representatives Skåne:<br>Karolin Isaksson<br>karolin.isaksson@med.lu.se<br>Bengt "Ahringberg-Kald<br>Bengt.Ahringberg-Kald@skane.se<br><br>Pathology representative Skåne:<br>Ingela Skogvall<br>Ingela.SkogvallSvensson@skane.se
Clinical monitoring organisation	Clinical trial unit:<br>Department of Clinical Cancer Studies<br>Eugeniavägen 6<br>Karolinska University Hospital<br>171 76 Stockholm
Statistics	Anders Berglund<br>Epistat AB<br>Dag Hammarskjölds väg 10C<br>752 37 Uppsala<br>Sweden<br>Telephone: +46 (0) 736-36 04 92<br>E-mail: anders.berglund@epistat.se

List of used acronyms and abbreviations

AE	Adverse Event, any untoward medical occurrence in a subject to whom a medicinal product is administered and which does not necessarily have a causal relationship with this treatment.
ALP	Alkaline Phosphatase
ALAT	Alanine Aminotransferase
ASAT	Aspartate Aminotransferase
BCR	B-cell Receptor
BRAF	Serine/threonine-protein kinase B-Raf
CLND	Compete lymph node dissection
CNS	Central Nervous System
CR	Complete Response
CRF	Case Report Form
CT	Computed Tomography
CTCAE	Common Terminology Criteria for Adverse Events
CTIS	Clinical Trials Information System = Centralized EU database/portal for application and communication with authorities concerning clinical trials. In Sweden this includes the Swedish Medical Products Agency and the Swedish Ethical Review Authority.
CTLA-4	Cytotoxic T-lymphocyte-Associated Protein 4
CTR	EU Regulation 536/2014, also called CTR, Clinical Trials Regulation
DMFS	Distant metastasis-free survival
DSMB	Data Safety Monitoring Board
DSUR	Development Safety Update Report = the standard which should be used for annual safety reporting to authorities
ECOG	Eastern Cooperative Oncology Group
eCRF	Electronical Case Report Form
EFS	Event-free survival
EMA	European Medicines Agency
EudraCT	European drug regulatory affairs Clinical Trials
FACS	Fluorescence Activated Cell Sorting

FDA	Food and Drug Administration
FNA	Fine-Needle Aspiration
GCP	Good Clinical Practice
GDPR	General Data Protection Regulation
IB	Investigator's Brochure
ICF	Informed Consent Form
ICI	Immune checkpoint inhibitors
IEC	Independent ethics committee
INMC	International Neoadjuvant Melanoma Consortium
INR	Index node resection
Ipi	Ipilimumab
irAE	immune-related adverse event
ITT	Intention-to-treat = including all data from all subjects who have participated in the trial
MEK	Mitogen-activated protein (MAP) kinase kinase
MPR	Major pathologic response (pathologic complete or near-complete response, ≤10% vital tumor cells found)
Nivo	Nivolumab
ORR	Overall Response Rate
OS	Overall Survival
PBMC	Peripheral Blood Mononuclear Cells
pCR	pathologic complete response
PD-1	Programmed cell Death protein 1
PET	Positron Emission Tomography
pnCR	Pathologic near-complete response (vital tumor cells found, but ≤10%)
pNR	Pathologic no response (>50% vital tumor cells found)
pPR	Pathologic partial response (>10%, but ≤50% vital tumor cells found)
PR	Partial Response
ProBNP	N-terminal pro b-type natriuretic peptide

RECIST	Response Evaluation Criteria In Solid Tumors
RFS	Recurrence-free survival
RR	Response Rate
SAE	Serious Adverse Event, Any untoward medical occurrence that at any dose requires inpatient hospitalization or prolongation of existing hospitalization, results in persistent or significant disability or incapacity, results in a congenital anomaly or birth defect, is life-threatening, or results in death
SAP	Statistical Analysis Plan
sEVs	Small Extra cellular Vesicles
SUSAR	Suspected Unexpected Serious Adverse Reaction. This is an event that is likely related to the investigational medicinal product but with unexpected occurrence. An adverse reaction is unexpected if its nature or seriousness is not consistent with the information on the product in the RSI.
TCR	T-cell Receptor
TLND	Therapeutic lymphadenectomy
WOCP	Women of childbearing potential

1. Synopsis

**EU CT number:** 2024-519593-39-00
**Title: SWE-NEO**: Swedish NeoAdjuvant Trial Comparing anti-PD-1 Monotherapy to Combined anti-CTLA-4/anti-PD-1 Blockade in Resectable Stage III Melanoma
**Trial ID:** SWE-NEO Trial
**Short background/ Rationale/Aim:**<br>At present two studies (SWOG S1801 and NADINA) have demonstrated superiority when using neoadjuvant treatment compared to adjuvant treatment only, but no studies have compared PD-1 monotherapy (SWOG 1801 regimen) to the PD-1/CTLA-4 combination (NADINA regimen) therapy. The SWE-NEO study aims to compare these two regimens, where the PD-1/CTLA-4 combination is potentially more effective, but also associated with more side effects.
**Study objectives and Endpoints:**<br>The primary objective is to study event-free survival (EFS) in patients with resectable stage III melanoma, receiving two different neoadjuvant immune checkpoint inhibitor (ICI) regimens, combined anti-CTLA-4/anti-PD-1 blockade or anti-PD-1 monotherapy. Secondary objectives include further efficacy and safety analysis. Exploratory objectives include biomarker analyses from sequential blood and tumor samples.<br><br><u>Primary endpoint:</u><br>• Event-free survival (EFS), defined as time from randomization to melanoma progression (irresectable stage III or stage IV disease), melanoma recurrence, or death from any cause (treatment-related, melanoma related or any other).<br><br><u>Secondary endpoints</u><br>• Relapse-free survival (RFS), defined as time between date of surgery and date of melanoma recurrence, treatment-related death or melanoma-related death, whichever occurs first.<br>• Distant metastasis-free survival (DMFS), defined as time between date of randomization and date of first distant metastasis, treatment-related death or melanoma-related death, whichever occurs first.<br>• Overall survival (OS), defined as time between date of randomization and date of death.<br>• Major pathological response (MPR) (≤10% viable tumor cells), difference in MPR between combined ICI and monotherapy, central review of all surgical specimens by three expert melanoma pathologists.<br>• Correlation of pathologic response in each arm to RFS, DMFS, and OS.

- Correlation of radiological and clinical response evaluation to RFS, DMFS, and OS.
- Proportion of patients having surgery according to plan (within 10 weeks from first neoadjuvant course).
- Surgical complication rates according to Clavien-Dindo surgical classification.
- Frequency and duration of all grade and grade 3-5 treatment-related adverse events (AEs) according to CTCAE 5.0.

Exploratory endpoints:

- Correlation of different biological markers analyzed from sequential blood and tumor samples with treatment efficacy and safety.

Trial design:

A phase III randomized controlled multicenter open-label trial. Patients will be randomized after a diagnose of resectable stage III melanoma to have either two courses of CTLA-4 and PD-1 inhibitor combination therapy or PD-1 inhibitor monotherapy, before the pre-planned operation. In both arms, adjuvant treatment with a PD-1 inhibitor or with BRAF+MEK inhibitors in patients with a BRAF V600E mutation, will be given only to patients with no major pathological response in the operated tumor, with PD-1 inhibitor, or with BRAF+MEK inhibitors in patients with BRAF V600E mutation. Active follow-up will be performed for 3 years from surgery and followed for survival up until 10 years. Sequential blood and tumor samples will be collected for biomarker analyses.

Trial population:

Patients 18 years or older with resectable stage III melanoma

Number of subjects:

128 patients, 64 + 64 patients in each arm. Patients that withdraw for any reason before starting neoadjuvant treatment will be replaced.

Inclusion criteria:

1. Participants must be at least 18 years of age.
2. Can provide a signed informed consent as described in the protocol, including compliance with the requirements and restrictions listed in the ICF and in this protocol.
3. World Health Organization (WHO) Performance Status 0 or 1.
4. Patients must have
   a. Histologically or cytologically confirmed Stage III melanoma. In the case of in-transit metastases (with or without lymph node metastases), ≤3 resectable in-transit metastases are allowed.
   b. Patients with cutaneous, acral, or unknown primary melanomas are eligible for enrollment.
   c. Resectable tumors are defined as having no significant vascular, neural or bony involvement. Only patients where a complete surgical resection with tumor-free margins can safely be achieved are defined as resectable.

5. Female patient of childbearing potential should have a negative urine or serum pregnancy test within 72 hours prior to receiving the first treatment. If the urine test is positive or cannot be confirmed as negative, a serum pregnancy test will be required.
6. Female patients of childbearing potential must be willing to use a highly effective method of contraception, for the course of the study through 150 days after the last dose of study medication. Note: Abstinence is acceptable if this is the usual lifestyle and preferred contraception for the subject. Highly effective methods of contraception include one or more of the following:
    a. male partner who is sterile (vasectomised) prior to the female study subject's entry into the study and is the sole sexual partner for the female subject;
    b. hormonal (oral, intravaginal, transdermal, implantable or injectable)
    c. an intrauterine hormone-releasing system (IUS)
    d. an intrauterine device (IUD) with a documented failure rate of < 1%.
7. Male patients of childbearing potential must agree to use an adequate method of contraception, starting with the first dose of study therapy through 150 days after the last dose of study therapy. Abstinence is acceptable if this is the usual lifestyle and preferred contraception for the subject. A unique female sexual partner must postmenopausal, permanently sterilized (e.g. hysterectomy or tubal ligation), or use a highly effective method of contraception.
8. No other malignancies, except if treated with curative intent and with a cancer-related life expectancy of more than 5 years.
9. No prior immunotherapy targeting CTLA-4, PD-1 or PD-L1.
10. No prior targeted therapy targeting BRAF and/or MEK.

Exclusion criteria:

1. Unresectable melanoma
2. Uveal/ocular or mucosal melanoma
3. Any serious or uncontrolled medical conditions that, in the investigator's opinion, may increase the risk associated with study participation or study drug administration, impair the ability of the subject to receive protocol therapy (including operation), or interfere with the interpretation of study results.
4. Subjects with a condition requiring systemic treatment with either corticosteroids (> 10 mg daily prednisone equivalents) or other immunosuppressive medications within 14 days of study drug administration. Inhaled or topical steroids and adrenal replacement doses > 10 mg daily prednisone equivalents are permitted in the absence of active autoimmune disease.
5. Women who are pregnant or breastfeeding.
6. Any condition that potentially hamper compliance with the study protocol and follow-up schedule; those conditions should be discussed with the subject before registration in the trial.

**Intervention, investigational medicinal products, dosage and administration:**<br><br>Patients will be randomized after a diagnose of resectable stage III melanoma to one of the following treatment arms:<br><br><u>Combination therapy group:</u> Patients will be treated with 2 cycles of intravenous infusion with ipilimumab 80 mg and nivolumab 240 mg followed by index node resection (if lymph node metastases) or radical surgery for other metastases. If major pathological response (MPR) occurs (<10% vital tumor cells), the patient receives no more treatment and is enrolled to follow-up. If there is no MPR, patients with lymph node metastases undergo therapeutic lymphadenectomy. Patients with no MPR then receive adjuvant treatment, nivolumab 480 mg (Q4w x12) or if BRAF V600E mutation, patients have 46 weeks daily tablets, dabrafenib 150 mg x2 and trametinib 2 mg 1x1.<br><br><u>Monotherapy group:</u> Patients will be treated with 2 cycles of intravenous infusion with nivolumab 480 mg followed by index node resection (if lymph node metastases) or radical surgery for other metastases. If major pathological response (MPR) occurs (<10% vital tumor cells), the patient receives no more treatment and is enrolled to follow-up. If there is no MPR, patients with lymph node metastases undergo therapeutic lymphadenectomy. Patients with no MPR then receive adjuvant treatment, nivolumab 480 mg (Q4w x12) or if BRAF V600E mutation, patients have 46 weeks daily tablets, dabrafenib 150 mg x2 and trametinib 2 mg 1x1.
**Ethical considerations, benefit/risk:**<br>Currently there are two approved types of neoadjuvant treatment in Sweden, with PD-1 inhibitor monotherapy (based on the SWOG S1801 study) or with a combination of PD-1 and CTLA-4 treatment (based on the NADINA study) starting approximately 1.5-2 months before the pre-planned surgery. The PD-1/CTLA-4 combination is potentially more effective, but also associated with more side effects and is a more expensive treatment. When presenting with resectable stage III melanoma, the acceptance of the risk of having serious, life-threating, or chronic side effects is generally lower than in the setting of inoperable metastatic disease. Hence, it is possible that the PD-1 monotherapy regimen is sufficient in this setting. It is therefore of essence to study this benefit/risk balance where the results can give valuable information what treatment is most beneficial, and the translational analyses aims to identify biomarkers that will help to choose the appropriate treatment for each patient.
**Planned duration of the trial:**<br>Q2/2025 – Q4/2032

2. Background and rationale

Despite improvements in the systemic treatment of malignant melanoma over the past decade, mainly through the introduction of immune checkpoint inhibitors (ICI) and targeted molecular therapy with BRAF and MEK inhibitors, morbidity and mortality for metastatic disease remains high (1). In macroscopic stage III melanoma, the primary tumor has spread to locoregional lymph nodes and tumor engagement of the lymph node is detectable on radiological and/or clinical examination. Resectable macroscopic stage III-IV disease mostly engages locoregional lymph node metastases but may also manifest as in-transit or satellite metastases or as oligometastatic resectable disease. Until recently, the standard of care for resectable stage III/IV disease was surgical removal of the tumor manifestation(s) followed by one year of adjuvant treatment with either monotherapy PD-1 inhibitor or BRAF/MEK inhibitors. However, the addition of adjuvant treatment fails to prevent recurrence of disease in almost half the patients (2). Due to this persistently high incidence of recurrent disease, during the past few years there has been a shift in focus from adjuvant treatment towards the potential benefit of neoadjuvant ICI treatment. The rationale behind a potential benefit of neoadjuvant ICI treatment lies in the increased availability immune cells in the tumor microenvironment and the higher presence tumor antigens in the neoadjuvant setting, as opposed to the their decreased availability following surgical resection of the tumor, as first shown in murine models of breast cancer (3).

A trial comparing single anti-PD1 therapy to dual ICI was published by Amaria et al. in 2018 (4). A dual ICI regimen of up to three neoadjuvant cycles of ipilimumab 3mg/kg + nivolumab 1mg/kg (ipi3/nivo1) was used. In the single anti-PD1 group, up to four cycles of nivolumab 3mg/kg was given). In total, 12 patients received single anti-PD1 therapy with nivolumab and 11 patients received dual ICI. In the dual ICI group, 45% of the patients achieved a complete pathologic response, as compared to 25% in the single nivolumab group. However, the combination therapy (in this study with high dose ipilimumab) was also associated with a high frequency of immune related side effects. In 2019, Rozeman et al published data from the OpACIN-neo trial (5). The authors aimed to evaluate the optimal dosing regimen of neoadjuvant immunotherapy for macroscopic stage III melanoma. In total, 86 patients were randomized to receive one of the three following neoadjuvant regimens: two cycles of ipi3/nivo1 q3w, two cycles of ipi1/nivo3 q3w, or two cycles of ipi3 q3w followed by two cycles of nivo3 q2w. Treatment-related adverse events (TRAEs) grade 3 or higher within the first 12 weeks were observed in 40% of the patients in the ipi3/nivo1 and in the ipi3 followed by nivo3 groups, and in 20% of the patients in ipi1/nivo3 group. Major pathologic response

(MPR), defined as ≤10% of viable tumor in the surgically resected specimen, was reported in 70% of patients in the ipi3/nivo1-group, 64% of patients in the ipi1/nivo3-group, and in 46% of patients in the ipi3 followed by nivo3-group. Hence, the OpACIN-neo trial showed that the regimen with two cycles of ipi1/nivo3 (i.e. the low-dose ipilimumab) was the best tolerated regimen with a high proportion of MPR and was therefore suggested to be a suitable regimen for neoadjuvant treatment.

Following the OpACIN-neo trial, Reijers et al. published data from the PRADO trial (6). In the PRADO trial, the pathologic response after neoadjuvant treatment with ipi1/nivo3 was evaluated by only assessing the largest lymph node (index lymph node, ILN) at baseline. This approach is in contrast to the therapeutic lymph node dissection (TLND) used in the OpACIN-neo trial, where all lymph nodes in the affected lymph node basin are removed. In the PRADO trial, patients with MPR in the ILN were not submitted to subsequent TLND, and did not receive adjuvant treatment. In patients who achieved partial pathological response in the ILN (>10% - ≤50% of viable tumor), a TLND was performed but no adjuvant treatment given. In patients with no pathological response in the ILN (>50% of viable tumor remaining), a TLND was performed and adjuvant treatment with either nivolumab or BRAF/MEK inhibitor (if the tumor had a BRAF V600E/K mutation) was administered with or without local radiotherapy.

In total, 99 patients were included. Within the first 12 weeks, TRAEs grade 3 or higher occurred in 22% of patients. Of the patients who underwent ILN resection, 61% had a MPR (12% pathologic complete response and 48% near-pathologic complete response) and an estimated recurrence-free survival (RFS) at 24 months of 93%. The RFS rates in patients with partial pathologic response and pathologic non-response were 64% and 71%, respectively.

In March of 2023, Patel et al. from the Southwest Oncology Group (SWOG) published the SWOG S1801 trial comparing neoadjuvant–adjuvant or adjuvant-only pembrolizumab in patients with clinically detectable stage III or resectable stage IV melanoma (7). Patients were randomized to treatment with either three cycles of neoadjuvant pembrolizumab followed by surgery and 15 cycles of adjuvant pembrolizumab (neoadjuvant-adjuvant group), or surgery without neoadjuvant treatment followed by 18 cycles of adjuvant pembrolizumab (adjuvant group). The main outcome of event-free survival (EFS) was a composite outcome and included the following events: disease progression or toxic effects of treatment that precluded surgery, inability to resect all gross disease, disease progression, surgical complications or toxic effects of treatment that precluded the initiation of adjuvant therapy within 84 days after surgery,

recurrence of melanoma after surgery, or death from any cause. In total, 154 patients were randomized to the neoadjuvant-adjuvant group and 159 patients to the adjuvant group. Of the 152 patients that received neoadjuvant treatment, 11 (7%) had at least one TRAE grade 3 or higher. The estimated EFS at two years was 72% in the neoadjuvant-adjuvant group and 49% in the adjuvant group. In a follow-up analysis (ESMO23) of pathologic responses in the neoadjuvant-adjuvant group, 53% of patients who underwent lymph node-dissection had a major pathological response (38% pathologic complete response and 15% pathologic near complete response).

In June 2024, Blank et al. published data from the NADINA trial (8), comparing two cycles of neoadjuvant ipi 80mg in combination with nivo 240mg q3w followed by surgery and then a pathologic response-driven treatment strategy, of either follow-up only in patients achieving MPR or adjuvant treatment (if no-MPR) with 11 cycles of 480 nivolumab q4w or BAF/MEK inhibitors for 46 weeks. The primary outcome was EFS, defined as the time from randomization to irresectable disease progression, disease recurrence, or death (due to melanoma or treatment). In total, 212 patients were randomized to the neoadjuvant group, and 211 patients were randomized to the adjuvant group. Of the 198 patients in the neoadjuvant group that underwent surgery, 119 (60%) had a MPR. TRAEs grade 3 or higher occurred in almost a third (29.7%) of the patients in the neoadjuvant group and in 14.7% of the patients in the adjuvant group. The estimated EFS at 12 months was 84% in the neoadjuvant and 57% in adjuvant groups respectively. An updated analysis of the trial showed an estimated EFS at 18 months of 81% in the neoadjuvant group and of 54% in the adjuvant group (9).

In summary, neoadjuvant treatment with single anti-PD1 therapy and neoadjuvant treatment with a combination of anti-PD1 therapy and anti-CTLA4 therapy have been studied in several pivotal trials, with results indicating a slightly higher rate of major pathologic response with dual ICI. However, no head-to-head randomized trial of single anti-PD1 compared to dual ICI has been performed. Given the above-described results from previous trials of neoadjuvant immunotherapy in stage III and IV resectable macroscopic melanoma, it is still unclear if neoadjuvant treatment with dual ICI confers a meaningful clinical benefit. This question is especially important in the context of the increased toxicity from dual ICI compared to single anti-PD1 therapy, with previous studies showing higher rates of grade 3 or higher TRAEs in approximately one third of patients receiving dual ICI. In the absence of such a trial, it is not possible to assess with sufficient certainty whether neoadjuvant dual ICI in resectable macroscopic stage III/stage IV carries an unnecessarily high risk of toxicity

compared to single anti-PD1 therapy, or if it is the optimal approach to improve survival outcomes.

To address these questions, we will perform a national, multi-center, randomized, clinical trial comparing dual ICI therapy to single anti-PD1 neoadjuvant therapy. Regimens already approved for neoadjuvant treatment in macroscopic resectable stage III-IV melanoma will be used. Patients will be randomized 1:1, to either two cycles of ipilimumab 80 mg + nivolumab 240 mg q3w or two cycles of nivolumab 480 mg q4w. After receiving neoadjuvant therapy, patients with lymph node metastases will undergo index node resection (INR), after previous (baseline) marking of the largest nodal metastasis. Pathologic response, as described by INMC, will be assessed in the index node. Patients with metastases other than lymph nodes will undergo radical surgery for their clinical metastases (in-transit, satellite or other metastases). Patients who achieved a major pathological response will not receive any further adjuvant treatment but will continue follow-up within the trial. In patients where an INR was performed, and who did not achieve major pathological response, they will undergo a TLND and receive 10 months of adjuvant nivolumab, or 46 weeks of dabrafenib+trametinib inhibitors if a BRAF V600E mutation is present.

The rationale for avoiding further treatment in patient achieving major pathological response stems from previous studies indicating a strong correlation between pathological response and long-term outcomes. An updated analysis of the NADINA trial, where patients who achieved major pathological response did not receive any adjuvant treatment, showed an estimated recurrence free survival of 93% at 18 months in patients who achieved major pathological response, as compared to 80% in partial responders and 61% in non-responders (9). If appropriate, omission of adjuvant treatment in patients with major pathological response would significantly reduce the risk of treatment related toxicities and healthcare burden. In the Swedish National care guidelines for melanoma, it is recommended to omit adjuvant treatment in the setting of major pathological response, irrespective of the neoadjuvant regimen received. The rationale for starting with INR instead of TLND as surgical method after neoadjuvant treatment is supported by the PRADO trial, where only INR was performed in patients who achieved major pathological response (6). As in the NADINA trial, major pathological response after neoadjuvant treatment in the PRADO trial was found to correlate strongly to event-free survival, which was estimated to 93% in patients who achieved a major pathological response (6).

3. Benefit-risk evaluation

In patients with resectable stage III melanoma, neoadjuvant treatment with ICI has shown significantly improved EFS compared to only giving adjuvant treatment after surgery. At the ESMO meeting in September 2022, the results from the phase II SWOG S1801 study were presented, demonstrating significantly improved EFS in the neoadjuvant compared to the adjuvant arm. Following the presentation of these findings, neoadjuvant treatment with PD-1 inhibitor monotherapy was implemented on a national level in Sweden, and this was initiated already in October 2022. As of November 2024, more than 300 patients have been treated with neoadjuvant treatment, the majority with nivolumab monotherapy (480 mg flat dose or 6 mg/kg, 2 courses with 4 weeks interval). Subsequently, at the ASCO meeting in June 2024, the results from the phase III NADINA study were presented, also demonstrating improved EFS in patients receiving neoadjuvant ipi1/nivo3. At present there are hence two studies demonstrating superiority when using neoadjuvant compared to adjuvant treatment only, but no studies that have compared neoadjuvant PD-1 monotherapy to PD-1/CTLA-4 combination therapy. Whilst it is important to be aware of possible bias when comparing the results from different studies, **Table 1** summarizes the main outcomes that have been published from both trials. Notably, the profile of the included patients was not entirely the same, in NADINA, all patients had lymph node metastasis and up to three in-transit metastases where allowed. In SWOG S1801 patients could have lymph node metastases or in-transit metastases but also resectable oligometastatic disease (M1a, M1b or M1c). In the table, percentages shown in green color indicates that a factor was numerically more favorable in one or the other study. Firstly, the radiological response rate was higher in the SWOG S1801 study. Secondly, there were more patients in the NADINA study that did not undergo surgery due to ICI related serious adverse events (SAEs), but also more patients in the SWOG 1801 that did not undergo surgery due to progression, possibly related to that this study included patients with more advanced disease. Thirdly a higher portion in the NADINA trial had an MPR, while there were fewer patients in SWOG S1801 that had pNR. Fourthly, the EFS at two years was marginally higher in the NADINA trial, however the fraction of patients with grade 3 AEs was considerably higher. A possible factor affecting the radiological response, operability and pathological response is the timing of the surgery, that in NADINA was earlier (after 2 cycles ICI) than in SWOG S1801 (after 3 cycles ICI). Regarding the operability, there is a certain risk of missing the “window of opportunity” in those not responding to ICI and the present experience is in favor of performing surgery approximately 45 days after start of ICI, as was done in the NADINA trial.

Table 1.

Comparision of design and main outcomes in the neoadjuvant arms of the NADINA and the SWOG 1801 studies

Study:	Study Phase	Neo-adj arm, n	Inclusion profile	Radiological response CR-PR	SD	PD	No surgery due to PD	SAE	Neo-1 => Operation	Pathological response MPR	pPR	pNR	Event-free survival (2y)	ICI related Gr ≥3 AEs
**NADINA** IPI/NIVO 80/240 mg Q3w x2	III	212	LN (3 IT allowed)	38%	45%	17%	2.4%	1.4%	45 days	63%	9%	28%	79%	30%
**SWOG 1801** PEMBRO 200 mg Q3w x3	II	154	LN or IT or oligo	50%	38%	12%	7.8%	0.6%	55 days	52%	29%	19%	72%	7%

Green font is to indicate that an outcome is more favorable in one or the other study
LN=Lymph node, IT=IN transit, oligo=oligometastatic disease (M1a, M1b or M1c)

In the SWE-NEO trial, combination ICI with ipi1/nivo3 will be compared to PD-1 inhibitor monotherapy, both arms will be given two cycles of treatment before the planned surgery. As has been described, there are no studies that have done such a comparison and the results from the two studies, NADINA and SWOG S1801, indicate that there are certain pros and cons of each treatment. The combination therapy can give a marginally improved efficacy, but at the same time the treatment has considerably more side effect and is also a more expensive treatment. When presenting with resectable stage III melanoma, the acceptance of the risk of having serious, life-threating, or chronic side effects is generally lower than in the setting of inoperable metastatic disease. Hence, it is possible that the PD-1 monotherapy regimen is sufficient in this setting. It is therefore of essence to study this benefit/risk balance where the results can give valuable information what treatment is most beneficial, and the translational analyses aims to identify biomarkers that will help to choose the appropriate treatment for each patient.

4. Trial objectives

4.1. Primary objective

The primary objective is to assess event-free survival (EFS) in patients with resectable stage III melanoma, comparing two different neoadjuvant ICI regimen, anti-PD-1 monotherapy or combined anti-CTLA-4/anti-PD-1 blockade.

4.2. Secondary objectives

Secondary objectives include further efficacy and safety analysis, including RFS, DMFS, OS and MPR (see section 4.5).

4.3. Exploratory objectives

Exploratory objectives include biomarker analyses from sequential blood and tumor samples.

4.4. Primary endpoint

Event-free survival (EFS), defined as time from randomization to melanoma progression (irresectable stage III or stage IV disease), melanoma recurrence, or death from any cause (treatment-related, melanoma related or any other).

4.5. Secondary endpoints

The secondary endpoints of this trial are:

- Relapse-free survival (RFS), defined as time between date of surgery and date of melanoma recurrence, treatment-related death or melanoma-related death, whichever occurs first.
- Distant metastasis-free survival (DMFS), defined as time between date of randomization and date of first distant metastasis, treatment-related death or melanoma-related death, whichever occurs first.
- Overall survival (OS), defined as time between date of randomization and date of death.
- Major pathological response (MPR) (≤10% viable tumor cells), difference in MPR between combined ICI and monotherapy, central review of all surgical specimens by three expert melanoma pathologists.
- Correlation of pathologic response in each arm to RFS, DMFS, and OS.
- Correlation of radiological and clinical response evaluation to RFS, DMFS, and OS.
- Proportion of patients having surgery according to plan (within 10 weeks from first neoadjuvant course).
- Surgical complication rates according to Clavien-Dindo surgical classification.
- Frequency and duration of all grade and grade 3-5 treatment-related adverse events (AEs) according to CTCAE 5.0.

4.6. Exploratory endpoints

The explorative endpoints of this trial are:

- Correlation of different biological markers analyzed from sequential blood and tumor samples with treatment efficacy and safety.

5. Trial design and procedures

5.1. Overall trial design

The SWE-NEO trial is a phase III randomized controlled multicenter open-label trial. Patients, 18 years or older will be randomized 1:1 after a diagnose of resectable stage III melanoma to have either two courses of ipi1/nivo3 or PD-1 inhibitor monotherapy, before undergoing surgery. In both arms, adjuvant treatment will be given only to patients with no major pathological response in the surgical specimen, with PD-1 inhibitor, or with BRAF+MEK inhibitors in patients with BRAF V600E mutation. 128 patients are planned to be included in the SWE-NEO trial, 64 in each arm. Patients with primary cutaneous, acral or unknown primary melanoma are eligible, while patients with uveal or mucosal melanoma are not. Active follow-up will be performed for 3 years from randomization. Sequential blood and tumor samples are collected for biomarker analyses.

In the combination therapy group patients will be treated with 2 cycles of intravenous infusion with ipilimumab 80 mg and nivolumab 240 mg q3w. In the monotherapy group patients will be treated with 2 cycles of intravenous infusion with nivolumab 480 mg q4w. After this treatment, patients with lymph node metastases will undergo index node resection, and patients with other type of metastases will undergo radical surgery. If major pathological response (MPR) occurs (<10% vital tumor cells), the patient receives no more treatment and is enrolled to follow-up. If there is no MPR, patients with lymph node metastases undergo therapeutic lymphadenectomy, and all patients then receive adjuvant treatment, if a BRAF V600E mutation is present, patients receive 46 weeks of dabrafenib 150 mg x2 and trametinib 2 mg 1x1.

5.2. Procedures and flow chart

SWE-NEO: Swedish Neoadjuvant Study Comparing anti-PD-1 Monotherapy to combined anti-CTLA-4/anti-PD-1 blockade in Resectable Stage III Melanoma

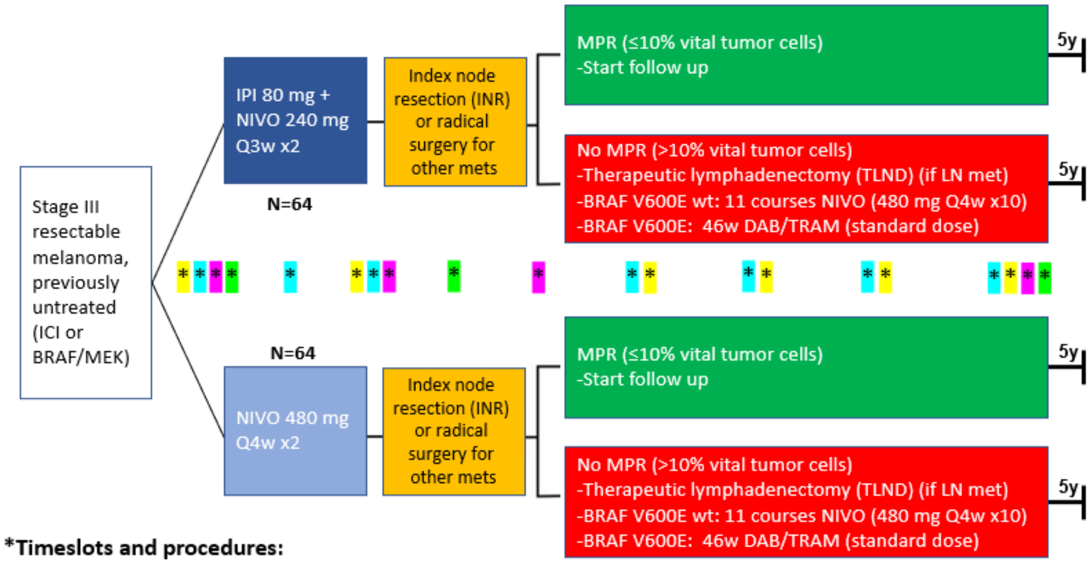

***Timeslots and procedures:**

<u>Randomization to Neo-adj-1: 0-6w</u>
-(PET)-CT (always including brain CT or separate brain MRI)
-Routine Lab
-Bloods for research
-Tumor biopsy for research

<u>NeoAdj-1 to INR or radical surgery: 6-10w (-14w)</u>
-(PET)-CT within 6-10w after Neo-Adj-1
-Routine Lab before Neo-Adj-2 and before op
-Bloods for research within 6-10w after Neo-Adj-1
-Operating material for research

<u>1st year after INR/TLND</u>
- INR/TLND (last operation) to Adj-1: 4-12w (if no MPR)
-(PET)-CT every 3rd month from INR/TLND
-Routine Lab before adjuvant courses and every 3rd month if in follow-up
-Bloods for research 1m after last operation

<u>Year 2-3 after INR/TLND</u>
-(PET)-CT every 6th month

<u>Year 4-5 after INR/TLND</u>
-Yearly check in medical chart for event including survival or relapse

<u>Tumor progression or relapse (0-5 ys)</u>
-Bloods for research at progression/relapse
-Tumor material at progression/relapse for research

Table 2. Visit schedule

	**Screening**	**Neoadjuvant treatment (N)**				**Surgery (S)**				**Adjuvant treatment/Follow-up**	**Progression or relapse/ End of Study**
		N1	Visit	N2	Visit	S1	Visit	S2	Visit	Visits after surgery (last surgery is Day 0)	
**Time**	–6w to –1d	0	0-5d before N2	21/28d	1-21d before S1	6-10w (max 14w)	1-8w after S1	1-10w after S1	1-9w after S2	3m, 6m, 9m, 12m, 18m, 24m, 30m, 36m	0–5 years
**Eligibility assessments**											
Informed Consent[1]	X										
Inclusion/Exclusion Criteria	X										
Medical history, melanoma[2] and other pre-existing conditions	X										
**Clinical Procedures**											
ECOG performance status	X		X				X		X	X	X
Clinical examination[3]	X		X				X		X	X	X
ECG	X										
**Study Treatments**											
Marking of index node[4]	(X)										
Neoadjuvant course 1[5]		X									
Neoadjuvant course 2[5]				X							
Surgery[6]						X					
TLND if no MPR in the index node[7]								(X)			
Adjuvant treatment (Nivolumab or BRAF/MEK)[8]										(X)	

	Screening	Neoadjuvant treatment (N)				Surgery (S)				Adjuvant treatment/Follow-up	Progression or relapse/ End of Study
		N1	Visit	N2	Visit	S1	Visit	S2	Visit	Visits after surgery (last surgery is Day 0)	
**Time**	–6w to –1d	0	0-5d before N2	21/28d	1-21d before S1	6-10w (max 14w)	1-8w after S1	1-10w after S1	1-9w after S2	3m, 6m, 9m, 12m, 18m, 24m, 30m, 36m	0–5 years
**Study Visits**											
Visit before neoadjuvant course 2[9]			X								
Visit before Surgery[10]					X						
Visit after Surgery[11]							X				
Visit after TLND[12]									(X)		
**Radiology**											
(PET)-CT neck, thorax, abdomen, brain (or brain MRI)[13]	X				X					X	X
**Laboratory Tests (clinical routine)**											
Serum and Plasma Chemistry[14]	X		X		X		X		X	X	X
CBC, differential count[15]	X		X		X		X		X	X	X
Pregnancy test (WOCBP only)[16]	(X)		(X)		(X)		(X)		(X)	(X)	
**Research tests**											
Blood for study purpose[17]	X				X				X		X
Tumor sampling for research[18]	X					X					X
**Adverse Events**											
AEs and SAEs [19]		Continuously									

[1]Written consent must be obtained by a physician study investigator prior to any protocol specific procedures. Results of tests performed in routine clinical management before informed consent is obtained, may be used for screening purposes if performed within the specified time limit (6 weeks).

[2]Medical history includes primary melanoma site and subtype, ulceration and BRAF status, number and type of metastasis (lymph node, in-transit etc.) and if it is synchronous or metachronous. The patients should be assessed at multidisciplinary conference to be eligible for the neoadjuvant procedure.
[3]Clinical examination includes assessment of clinical status, including ECOG. . If palpable or visible tumor, a description of the location and size of the metastasis/es should be noted.
[4]For patients with lymph nodal metastases, a marker (according to local routine) will be placed in the largest metastatic node (index node) by ultrasound for later index node resection (INR).
[5]Neoadjuvant treatment with ipilimumab 80 mg and nivolumab 240 mg in the combination arm (Q3x2) and nivolumab 480in the monotherapy arm (Q4x2).
[6]Index node resection (INR) if lymph node metastasis/es or radical surgery for other metastasis/es, 6-10w after baseline (allowed until 14w, but at 11-14w reported as "surgery not on time")
[7]Therapeutic lymphadenectomy (TLND) if patient has no major pathological response (MPR) in the index node (not applicable in patients with in-transit metastases only)
[8]Adjuvant treatment only if no MPR, nivolumab 480 mg (Q4 x11) if BRAF V600E wt and for BRAF V600E mutated, dabrafenib (daily 150 mg x2) and trametinib (daily 2 mg x2) for 46 weeks. Adjuvant treatment should start at the latest 12 weeks after the last operation.
[9]Visit before neoadjuvant course 1 includes assessment if clinical status and laboratory tests are acceptable for second neoadjuvant course. If palpable or visible tumor, description of the location and size of the metastasis/es and any clinically notable change compared to the baseline. Ensure that radiological exam after N2 is booked as well as date for operation.
[10]Visit before planned surgery includes assessment if clinical status, radiology and laboratory tests are acceptable for operation. If palpable or visible tumor, description of the location and size of the metastasis/es and any clinically notable change compared to the baseline. If apparent progressive disease reevaluation regarding operability at the multidisciplinary conference.
[11]Visit after surgery includes review of pathology report from operation, if MPR patient starts follow-up. If no MPR in patient operated with INR, TLND should be planned. Patients with no MPR operated radically for other metastases start adjuvant treatment (see nr. 7). Notification of surgical complications according to the Clavien-Dindo Classification. Assessment if clinical status and laboratory tests are acceptable for adjuvant therapy or TLND.
[12]Visit after TLND includes notification of surgical complications according to the Clavien-Dindo Classification. Assessment if clinical status and laboratory tests are acceptable for adjuvant therapy (see nr. 7).
[13]Contrast-enhanced computed tomography (CT) of the chest, abdomen and other known/suspected sites of disease, and brain CT or MRI. At screening and before the planned operation measurement according to RECIST 1.1 criteria. Measurable disease by RECIST 1.1 is however not an inclusion criterion, only pathologically verified resectable stage III disease. Measurement (in mm) of all visible tumors at baseline and before surgery. Specification of any new metastatic lesions compared to baseline.

[14]Serum and plasma chemistry includes analysis of AST, ALT, ALP, Bilirubin, LDH, Creatinine, Na, K, Glucose, TSH, T3, T4, Cortisol, Lipase, Amylase, CRP, Albumin, Troponin-T and pro-BNP. Will be performed by local study site or their contract laboratory. Performed at screening/baseline, prior to all oncological treatments and follow up visits.
[15]Circulating blood cells (CBC) includes Hb, thrombocyte, leukocyte count and a differential count. Will be performed by local study site or their contract laboratory. Performed at screening/baseline, prior to all oncological treatments and follow up visits.
[16]For women of childbearing potential (WOCBP), a urine or serum pregnancy test will be performed within 72 hours before start of first study treatment and prior to all subsequent treatment visits during treatment phase. After end of study treatment pregnancy testing should continue at monthly intervals until approximately 5 months after the last given dose of study treatment. If the urine test is positive or cannot be confirmed as negative, a serum pregnancy test will be required. The pregnancy test must be negative for the patient to be eligible.
[17]Blood samples for research include 3x 10ml BD Vacutainer EDTA tubes for plasma. Additionally, 10x 9mL Na-Hep tubes for peripheral blood mononuclear cells (PBMC) should be obtained. For details, see 5.3 Biological sampling procedures.
[18]Biopsies of the tumor taken during the screening period, part of the excised tumor (after the neoadjuvant treatment) and biopsy or surgical material from tumor at progression or relapse. For details, see 5.3 Biological sampling procedures.
[19]Patient are followed continuously for adverse events (AEs) and serios adverse events (SAEs), from start of neoadjuvant study treatment and 365 days after last treatment (ICI, surgery or BRAF/MEK).

5.3. Biological sampling procedures

5.3.1. Handling, storage, and destruction of biological samples

Blood samples are collected at up to four time points, at screening, before and after surgery and in the case of progression or relapse (see flow chart 5.2). Plasma samples will be obtained at each time point using 3x10 mL BD Vacutainer EDTA 10 tubes. At the same time points PBMC will be obtained from 10x10 mL in Na-heparin coated vacutainer tubes. Samples for plasma and PBMC are collected by research nurses and sent to responsible team/scientist at each site. The blood samples will be cryopreserved and stored within the Regional Biobanks. Protocols for handling/freezing of plasma and PBMC will be shared and are the same among sites. PBMCs will be analyzed by flow cytometry analysis (FACS) of different immune cell populations. Single cell RNA, TCR and BCR sequencing for deeper analysis of peripheral blood will be performed using the 10x Genomics Chromium X technology. Extracellular vesicle (EV) protein and RNA as well as circulating tumor DNA (ctDNA) analysis will be performed on plasma samples using platforms such as Olink.

Tumor samples will be collected before and after the neoadjuvant therapy and in the case of progression or relapse (see flow chart 5.2). Two core biopsies will be taken before neoadjuvant treatment. One tumor biopsy will immediately be put in formalin for histological and spatial transcriptomics analyses. The other biopsy will be processed at each site and stored as viable tissue for further analysis. Surgical tissue after neoadjuvant treatment is also obtained for research. From all patients, formalin-fixed and paraffin embedded operating material is required, in the form of blocks from part of the excised tumor that will be used for research. In the case of relapse two core biopsies (or surgical material) will be taken and processed and stored at respective site, one in formalin and the other is stored as viable tissue. Tissue samples should be collected instantly by research nurses and sent to responsible pathologists/scientist at each site where they will be processed. The tumor samples are stored within the Regional Biobanks. TIL extraction from tissue will be performed by responsible scientist at the respective site. Protocols for handling viable tissue and extraction of TILs will be shared between sites. Single cells from tumor tissue will be subjected to single cell RNA, TCR, BCR sequencing using the 10xGenomics Chromium X technology. Moreover, spatial transcriptomics and multiplex immunostaining will be performed on all tissue samples and will be analyzed using the Xenium system.

In the information provided to subjects, subjects will be fully informed about how their samples will be collected, used and disclosed. The content of the informed consent form complies with relevant integrity and data protection legislation. The

subject information and the informed consent form will explain how trial data are stored to maintain confidentiality in accordance with national data legislation. All information will be pseudonymized and identified with Trial code/Trial ID/Initials.

5.3.2. Total volume of blood per subject

The total volume of blood taken from each subject during the trial is a maximum of 520 ml, over 36 months.

Plasma: 4 x 3 x 10ml =120ml

PBMC: 4 x 10 x 10ml =400ml

5.3.3. Biobank

All research samples taken in this study are released to Stockholm Medical Biobank (IVO reg. no 914) and handled according to the current biobank laws and regulations. The Biobank Act (2023:38) is not applicable for the samples for pregnancy test and clinical laboratory assessments since these samples are not stored but are destroyed immediately after the analysis. All biobank samples are pseudonymized to protect the subject’s identification. All samples and the identification/code list are stored securely and separately to prevent unauthorized access.

All analyses will be performed at laboratories at Karolinska Institute, Karolinska University Hospital, Gothenburg University and Lund University. In any case samples are sent, a Material Transfer Agreements (MTA) between involved parties will be established.

Samples will be stored for up to 20 years after the end of study, after which they will be destroyed.

The study subjects have the right to withdraw their study specific samples and have them destroyed but results already obtained from the samples will be used in the evaluation of the study. Following a request from a subject to withdraw samples, the investigator is to provide the sponsor with the study subject number so that any remaining tissue samples can be located and destroyed. Additional analysis beyond the scope of this study must be approved by the Sponsor and the Swedish Ethical Review Authority.

5.4. Start, end, temporary halt and early termination

5.4.1. Start of the clinical trial

The clinical trial will not commence until clinical trial approval is in place.

5.4.2. Temporary halt or early termination

The study may be temporarily halted or prematurely terminated due to safety aspects, such as an unacceptable number of serious adverse events (SAE). If recruitment of subjects cannot be met within reasonable time limits the trial may be prematurely terminated. If the study is prematurely terminated or temporarily halted, the investigator should immediately inform the subjects about this and ensure appropriate treatment and follow-up. The regulatory authority should be informed as soon as possible, but no later than within 15 days. Decisions on a temporary halt or an early termination are taken by the sponsor.

5.4.3. End of the clinical trial

The end of trial is defined as date of the last visit of the last subject participating in the study and is anticipated Q4 2032.

6. Subject selection

Resectable stage III cutaneous, acral or unknown primary melanoma patients found at a multidisciplinary conference to be candidate for the neoadjuvant treatment procedure. Patients need to be naïve for CTLA-4/PD-1/PD-L1 blockade and BRAF + MEK inhibition, and at least 18 years of age.

6.1. Inclusion criteria

1. Participants must be at least 18 years of age.
2. Can provide a signed informed consent as described in the protocol, including compliance with the requirements and restrictions listed in the ICF and in this protocol.
3. World Health Organization (WHO) Performance Status 0 or 1.
4. Patients must have

   a. Histologically or cytologically confirmed Stage III melanoma. In the case of in-transit metastases (with or without lymph node metastases), ≤3 resectable in-transit metastases are allowed.
   b. Patients with cutaneous, acral, or unknown primary melanomas are eligible for enrollment.
   c. Resectable tumors are defined as having no significant vascular, neural or bony involvement. Only patients where a complete surgical resection with tumor-free margins can safely be achieved are defined as resectable.
5. Female patient of childbearing potential should have a negative urine or serum pregnancy test within 72 hours prior to receiving the first treatment. If the urine test is positive or cannot be confirmed as negative, a serum pregnancy test will be required.
6. Female patients of childbearing potential must be willing to use a highly effective method of contraception, for the course of the study through 150 days after the last dose of study medication. Note: Abstinence is acceptable if this is the usual lifestyle and preferred contraception for the subject. Highly effective methods of contraception include one or more of the following:
   a. male partner who is sterile (vasectomised) prior to the female study subject’s entry into the study and is the sole sexual partner for the female subject;
   b. hormonal (oral, intravaginal, transdermal, implantable or injectable)
   c. an intrauterine hormone-releasing system (IUS)
   d. an intrauterine device (IUD) with a documented failure rate of < 1%.
7. Male patients of childbearing potential must agree to use an adequate method of contraception, starting with the first dose of study therapy through 150 days after the last dose of study therapy. Abstinence is acceptable if this is the usual lifestyle and preferred contraception for the subject. A unique female sexual partner must postmenopausal, permanently sterilized (e.g. hysterectomy or tubal ligation), or use a highly effective method of contraception.
8. No other malignancies, except if treated with curative intent and with a cancer-related life expectancy of more than 5 years.
9. No prior immunotherapy targeting CTLA-4, PD-1 or PD-L1.
10. No prior targeted therapy targeting BRAF and/or MEK.

6.2. Exclusion criteria

1. Unresectable melanoma
2. Uveal/ocular or mucosal melanoma
3. Any serious or uncontrolled medical conditions that, in the investigator's opinion, may increase the risk associated with study participation or study drug administration, impair the ability of the subject to receive protocol therapy (including operation), or interfere with the interpretation of study results.
4. Subjects with a condition requiring systemic treatment with either corticosteroids (> 10 mg daily prednisone equivalents) or other immunosuppressive medications within 14 days of study drug administration. Inhaled or topical steroids and adrenal replacement doses > 10 mg daily prednisone equivalents are permitted in the absence of active autoimmune disease.
5. Women who are pregnant or breastfeeding.
6. Any condition that potentially hamper compliance with the study protocol and follow-up schedule; those conditions should be discussed with the subject before registration in the trial.

6.3. Screening and inclusion

The following screening procedures and tests will be completed during the screening phase within 6 weeks. The informed consent form needs to be signed prior to any study-specific screening procedures.

- Baseline (PET-)CT, including brain CT or separate brain MRI.
- Noting the size (maximum size in mm), and location of any lesions that exists.
- Noting of relevant medical history.
- Noting of ECOG performance status.
- ECG.
- Beta-HCG pregnancy test for WOCP.
- Routine lab hematologic parameters: Hb, platelet count, absolute neutrophil count (ANC), white blood cell differentiation.
- Routine lab chemistry: LDH, sodium, potassium, chloride, calcium, creatinine, albumin, ASAT, ALAT), bilirubin, gGT, ALP, glucose, amylase, lipase, CPK, TSH, fT4, ACTH, cortisol, CRP, Troponin T, proBNP, CK-MB, albumin, CRP.
- Tumor biopsy for research (see biological sampling procedures for details)
- Blood sampling for research (see biological sampling procedures for details)

6.4. Withdrawal criteria

A subject who has discontinued the trial before starting the study treatment (the first neoadjuvant course) can be replaced to achieve the desired number of included subjects.

Subjects can discontinue their participation in the trial at any time without any consequence to his/her continued treatment. The investigator/sponsor can at any time terminate the trial for a subject due to safety concerns e.g., unacceptable adverse events/adverse reactions or because the subject does not follow procedures in the clinical trial protocol. In either case, serious adverse events, disease progression and overall survival will be followed up.

If the subject discontinues the trial, follow-up of this subject will be performed according to the clinic's routine.

Discontinuation Criteria for Individual Subjects:

1. Inappropriate enrolment (violation of Inclusion / Exclusion Criteria)

2. Withdrawal of consent

3. Progression

4. Discontinuation of study drugs due to AE

5. Pregnancy

The reasons for discontinuation should be recorded in the electronic case report form (eCRF). Patients discontinued from the study will be asked to return for an "End of Treatment Visit" and will thereafter be taken care of and followed at the discretion of the treating physician.

After the 36 months of planned active follow-up, patients will be taken care of and followed at the discretion of the treating physician, additional follow-up for ongoing serious adverse events, disease progression and survival will continue.

7. Trial treatments

All the drugs in the SWE-NEO study are recommended in Sweden for the indication they will be used for in the study and administrated according to standard dosages. Administration, use and handlining of side effects is according to the Swedish national "Regime library": Nationellt regimbibliotek för cancerläkemedel - RCC

In the combination therapy group patients will be treated with 2 cycles of intravenous infusion with ipilimumab 80 mg and nivolumab 240 mg followed by index node resection for lymph node metastases and radical surgery for other metastases. If major pathological response (MPR) occurs (<10% vital tumor cells), the patient receives no more treatment and is enrolled to follow-up. If there is no MPR, patients with lymph node metastases undergo therapeutic lymphadenectomy. Patients with no MPR receive adjuvant treatment, if BRAF V600E mutation patients have 46 weeks daily tablets, dabrafenib 150 mg x2 and trametinib 2 mg 1x1.

In the monotherapy group patients will be treated with 2 cycles of intravenous infusion with nivolumab 480 mg followed by index node resection for lymph node metastases and radical surgery for other metastases. If major pathological response (MPR) occurs (<10% vital tumor cells), the patient receives no more treatment and is enrolled to follow-up. If there is no MPR, patients with lymph node metastases undergo therapeutic lymphadenectomy. Patients with no MPR receive adjuvant treatment, if BRAF V600E mutation patients have 46 weeks daily tablets, dabrafenib 150 mg x2 and trametinib 2 mg 1x1.

7.1. Description of investigational medicinal product(s)

All drugs in the SWE-NEO study will be used according to clinical routine and administrated according to standard dosages. Administration, use and handlining of side effects is according to the Swedish national “Regime library”: Malignt melanom - RCC Kunskapsbanken

Ipilimumab (Keytruda) manufactured by BMS is administrated intravenously. In the combination arm 80 mg are given as a 30-minute infusion at day 1 and day 21 as part of the neo-adjuvant regimen. The drug will be ordered from the hospital pharmacy. Treatments can be delayed or halted due to side effects or other health issues (as deemed by the treating physician), while dose reductions are not applied.

Nivolumab (Opdivo) manufactured by BMS is administrated intravenously. In the combination arm 240 mg are given as a 30-minute infusion at day 1 and day 21 as part of the neo-adjuvant regimen. In the monotherapy arm and also in the adjuvant setting, 480 mg are given as a 30-minute infusion with 4-week interval. The drug will be ordered from the hospital pharmacy. Treatments can be delayed or halted due to side effects or other health issues (as deemed by the treating physician), while dose reductions are not applied.

7.1.1. Dose and administration

Dosage and administration of nivolumab and ipilimumab, both in the combination and monotherapy arm, is according to clinical routine for the neoadjuvant and adjuvant treatment of melanoma. Both ipilimumab and nivolumab are monoclonal antibodies administrated as intravenous infusions.

7.1.2. Packaging, labelling, and handling of investigational medicinal products(s)

This is a study on already approved medicinal products for the indication used in the study, where the investigational drugs will be provided according to the clinical routine at each hospital. Patients receive appropriate information about the drugs, also according to the clinical routine at the hospitals that all have long-going experience in treating the patient group that will be included, with the drugs that are used in the study. Ipilimumab, nivolumab will be acquired from the hospital pharmacy at the time of use according to clinical routine at each hospital. There will hence be no specific labeling of the drugs.

7.1.3. Drug accountability and treatment compliance

Study treatment with nivolumab and ipilimumab is administered according to clinical routine at the clinic. Treatment with Dabrafenib and trametinib are also administered according to standard of care. Drug accountability and monitoring of treatment compliance is therefore not applicable.

7.1.4. Randomisation

Subjects are included/randomised consecutively as they are found to be eligible for inclusion in the trial. If a subject discontinues their participation, the subject's trial-specific code will not be reused, and the subject will not be allowed to re-enter the trial again. Randomisation will be carried out by use of the web-based randomisation system ALEA, provided by Clinical Trials Office, Center for Clinical Cancer Studies, Karolinska University Hospital.

7.1.5. Blinding

SWE-NEO is a non-blinded study.

7.1.6. Destruction

Investigational medicinal products will be destroyed according to clinical routine if necessary.

7.2. Auxiliary medicinal products

Dabrafenib (Tafinlar) manufactured by Pierre-Fabre is taken as tablets. In the adjuvant setting, patients take 75 mg tablets, 2 tablets twice a day. The drug will be prescribed by the doctor and patients get the tablets from the pharmacy. Dose adjustments, pauses and treatment halt due to side effects or other health issues are handled by the treating physician.

Trametinib (Mekinist) manufactured by Pierre-Fabre is taken as tablets. In the adjuvant setting, patients take 1 tablet daily. The drug will be prescribed by the doctor and patients get the tablets from the pharmacy. Dose adjustments, pauses and treatment halt due to side effects or other health issues are handled by the treating physician.

7.2.1. Dose and administration

Dabrafenib and trametinib are also administrated according to the standard approved dosage as adjuvant therapy in melanoma. Dabrafenib and trametinib are small molecule drugs taken daily, as tablets. Administration, use and handlining of side effects is according to the Swedish national “Regime library”: Malignt melanom - RCC Kunskapsbanken.

7.2.2. Drug accountability and treatment compliance

There are no auxiliary medical products in the study.
Treatment with Dabrafenib and trametinib are administered according to standard of care. The dabrafenib and trametinib tablets will be prescribed by the physician and collected by the patient that themselves take the tablets daily, also according to the clinical routine at each hospital. Drug accountability and monitoring of treatment compliance is therefore not applicable.

7.3. Concomitant use of other medicinal products and treatments

Medications considered necessary for the safety and well-being of the subject may be provided at the discretion of the investigators, unless otherwise specified in the exclusion criteria.

7.4. Treatment after trial end

Patients will be followed up by radiological exams and visits to the hospital until 3 years have passed from the surgery. In SWE-NEO, there will thereafter be a yearly check in the electronic medical record for survival or relapse up to 5 years. After the follow-up visits end, there is no planned treatment. Patients will at progression or relapse, at any time receive treatment based on the treating physicians' clinical recommendations.

8. Methods for measurement of endpoints for clinical efficacy and safety

8.1. Methods for measurement of endpoints for clinical efficacy

8.1.1. Primary endpoint

The primary end point is **Event-free survival (EFS).** EFS will be studied as time-to-event defined as the time from inclusion until (whatever occurs first):

- The date of first documented disease progression (defined as irresectable disease or the appearance of new radiologically pathologically verified metastases) during the neoadjuvant treatment.
- The date of radiologically or pathologically verified melanoma recurrence after the surgery.
- The date of death from any cause (treatment-related, melanoma related or any other).
- Census date.

8.1.2. Secondary endpoints

Relapse-free survival (RFS). RFS will be studied as time-to-event defined as the time from the last surgery until (whatever occurs first):

- The date of radiologically or pathologically verified melanoma recurrence after the surgery.
- The date of melanoma related death

- Census date.

Distant metastasis-free survival (DMFS). DMFS will be studied as time-to-event defined as the time from inclusion until (whatever occurs first):

- The date of radiologically or pathologically verified distant metastases.
- The date of melanoma related death
- Census date.

Overall survival (OS). OS will be studied as time-to-event defined as the time from inclusion until (whatever occurs first):

- The date of death from any cause (treatment-related, melanoma related or any other).
- Census date.

Major pathological response (MPR). The fraction of patients having MPR, defined as ≤10% vital tumor cells in the surgical sample will be compared between the combination therapy and monotherapy arms. There will be a central review of all samples by three expert melanoma pathologists.

Correlation of the pathological response to survival outcomes, RFS, DMFS and OS. Pathological response is defined as:

- Pathological complete response (pCR): 0% vital tumor cells in surgical sample
- Pathological near complete response (pnCR): 1-10% vital tumor cells in surgical sample
- Pathological partial response (pPR): 11-50% vital tumor cells in surgical sample
- No pathological response (pNR) >50% vital tumor cells in surgical sample

Correlation of radiological or clinical response evaluation to survival outcomes, EFS, RFS, DMFS, and OS. The radiology before start of the first neoadjuvant treatment is compared to the radiology 5-8w after start (when the patient should have received two neoadjuvant courses, or only one course in the case of side effects resulting in cessation of neoadjuvant treatment. The target lesion defined as the largest measurable lesion in the patient and the response is evaluated according to RECIST 1.1. as follows:

- Complete response (CR): Disappearance of the target lesion
- Partial response (PR): At least a 30% decrease in the size of the target lesion.
- Stable disease (SD): Neither sufficient shrinkage to qualify for PR nor sufficient increase to qualify for PD.
- Progressive disease (PD): At least a 20% increase in the size of the target lesions or the appearance of one or more new lesions.

For patients not having a lesion fulfilling the RECIST 1.1 criteria, such as in-transit or satellite metastases that are only clinically evaluable, lesions should be photographed with visible measurements and depending on the evolvement

defined by the investigator physician seeing the patient at the visit before the planned surgery, as CR, PR, SD and PD. Patients with pathologically verified lesions under 1 cm in largest diameter of largest lesion, can also be included and response should be clinically or radiologically defined as CR, PR, SD and PD defined with the same percentages as in the RECIST 1.1 evaluation for target lesions.

Rate having **surgery according to plan** (within 10w from first Neoadjuvant course). The rate not having surgery according to plan and the underlying reasons will be compared in the two treatment arms:

- Patients having the pre-planned surgery, within 10 weeks from the first neoadjuvant course are designated as *having had the surgery according to plan.*
- Patients having had their surgery in week 11-14, are designated as having had a *late surgery.* It should be stated if the late operation was due to side effects, tumor progression or other factors.
- Patients not operated or operated after 14 weeks are designated as *not having had the surgery as planned.* It should be stated if the reason for not having the pre-planned operation was due to side effects, tumor progression or other factors.

Surgical complication rates will be reported up 90 days after the final surgical procedure according to Clavien-Dindo surgical classification and the Comprehensive Complication Index (CCI).

The Clavien-Dindo classification:

Grade I — Any deviation from the normal postoperative course without the need for pharmacological treatment or surgical, endoscopic and radiological interventions. Allowed therapeutic regimens are: drugs as antiemetics, antipyretics, analgesics, diuretics and electrolytes and physiotherapy. This grade also includes wound infections opened at the bedside.

Grade II — Requiring pharmacological treatment with drugs other than such allowed for grade I complications. Blood transfusions and total parenteral nutrition are also included.

Grade III — Requiring surgical, endoscopic or radiological intervention. Not under general anesthesia (Grade IIIa) or under general anesthesia (Grade IIIb).

Grade IV — Life-threatening complication, including CNS complications (including brain hemorrhage, ischemic stroke and subarachnoid bleeding, but excluding transient ischemic attacks), requiring IC/ICU-

management. Single organ dysfunction (Grade IVa) or multi organ dysfunction (Grade IVb).

Grade V Death of patient.

The Comprehensive Complication Index (CCI) is indexed between 0-100 as a sum of all Clavien-Dindo complications in one patient weighted by their severity as calculated by:
$CCI = \frac{\sqrt{(wC_1 + wC_2 \ldots + wC_x)}}{2}$, where wC = Weight of Complication with wC Grade I = 300, wC Grade II = 1750, wC Grade IIIa = 2750, wC Grade IIIb = 4550, wC Grade IVa = 7200 and wC Grade IVb = 8550. Presence of a complication Grade V automatically results in CCI score 100.

Frequency and duration of all grade and grade 3-5 **treatment-related adverse events (AEs) according to CTCAE 5.0.**

- Patient are followed continuously for adverse events (AEs) and serios adverse events (SAEs), from start of neoadjuvant study treatment and 365 days after last treatment (ICI, surgery or BRAF/MEK) and

8.1.3. Exploratory endpoints

Correlation of different biological markers analyzed from sequential blood and tumor samples with treatment efficacy and safety. The following will be studied as potential biomarkers.

- Circulating tumor DNA (ctDNA) in plasma
- Extracellular vesicles (EVs) in plasma
- Fluorescence-activated Cell Sorting (FACS) to profile immune cells in peripheral blood mononuclear cells (PBMC) isolated from blood
- Genomic and expression analyses of tumors, including spatial and single cell analyses.
- Analyses of tumor infiltrating lymphocytes

8.2. Methods for measurement of endpoints for clinical safety

Safety assessments include AEs and SAEs, physical examinations, vital signs, performance status, assessment of signs and symptoms and laboratory tests as outlined in the Flow Chart. All clinical safety laboratory assessments will be performed locally as per the Flow Chart. Laboratory toxicities (e.g., suspected drug induced liver enzyme evaluations) will be monitored during the follow-up phase via on site/local labs until all study treatment-related toxicities resolve, return to baseline, or are deemed irreversible.

9. Handling of Adverse Events

Supportive Care Guidelines for Immune Related AEs (irAEs)

Subjects should receive appropriate supportive care measures as deemed necessary by the treating investigator as per local or international practice guidelines. Suggested supportive care measures for the management of adverse events with potential immunologic aetiology are outlined in the Swedish National Guidelines for the handling of irAEs. Bedömning och hantering av biverkningar vid behandling med checkpointhämmare - RCC Kunskapsbanken. Where appropriate, these guidelines include the use of oral or intravenous treatment with corticosteroids as well as additional anti-inflammatory agents if symptoms do not improve with administration of corticosteroids. Note that several courses of steroid tapering may be necessary as symptoms may worsen when the steroid dose is decreased. For each disorder, attempts should be made to rule out other causes such as metastatic disease or bacterial or viral infection, which might require additional supportive care. The treatment guidelines are intended to be applied when the investigator determines the events to be related to ipilimumab and/or nivolumab.

Common side effects from dabrafenib are trametinib are pyrexia, chills, headache, rash and gastrointestinal symptoms. The symptoms typically resolve within a few days if the patient stops taking the tablets. Symptoms are managed mainly by antipyretics and by pausing the drug or lowering the dose. The measures taken will be as deemed necessary by the treating investigator. Suggested supportive care measures for the management of adverse events from Dabrafenib and trametinib, including when to paus or adjust the dose, is outlined in the Swedish national “Regime library”: Malignt melanom - RCC Kunskapsbanken

9.1. Definitions

9.1.1. Adverse Event (AE)

Adverse Event (AE): Any untoward medical occurrence in a subject to whom a medicinal product is administered and which does not necessarily have a causal relationship with this treatment.

9.1.2. Adverse Reaction (AR)

All noxious and unintended reactions to the medicinal product related to any dose should be considered an adverse reaction (AR). The phrase “reaction” to a medicinal product means that the causal relationship between the medical product and an adverse event is at least a reasonable possibility, that is the relationship cannot be ruled out.

9.1.3. Serious Adverse Event (SAE)

Serious Adverse Event (SAE): Any untoward medical occurrence that at any dose requires inpatient hospitalization or prolongation of existing hospitalisation, results in persistent or significant disability or incapacity, results in a congenital anomaly or birth defect, is life-threatening, or results in death.

Medical and scientific assessment will be made to determine if an event is serious.

9.1.4. Suspected Unexpected Serious Adverse Reaction (SUSAR)

SUSAR: An adverse reaction/event that is unexpected, serious, and suspected to be caused by the treatment, i.e. adverse reactions/events that are not included in the RSI section of the Investigator’s Brochure (IB) or SmPC.

9.2. Assessment of Adverse Events (AE)

9.2.1. Assessment of causal relationship

The investigator is responsible for determining whether there is a causal relationship between the AE/SAE and use of the investigational medicinal product.

Consideration should be given to whether there is a reasonable possibility of establishing a causal relationship between the adverse event and the investigational medicinal product based on the analysis of the available evidence.

All AE can be categorized as either likely related, possibly related, unlikely related or not related, in accordance with the definitions below:

Likely related: Clinical event, including abnormal results from laboratory analyses, occurring within a reasonable time after administration of the

intervention/investigational medicinal product. It is unlikely that the event can be attributed to underlying disease or other medications but is most likely caused by the investigational medicinal product and its emergence is reasonable in relationship with use of the investigational medicinal product.

Possibly related: Clinical event, including abnormal results from laboratory analyses, occurring within a reasonable time after administration of the intervention/investigational medicinal product. The event could be explained by the investigational medicinal product and its emergence is reasonable in relationship with use of the investigational medicinal product, but there is insufficient information to determine the relationship. The event could be explained by an underlying disease or other medications.

Unlikely related: Clinical event, including abnormal responses from laboratory tests, unlikely to be related to the intervention/investigational medicinal product and can be reasonably explained by other medication or underlying disease.

Not related: Clinical event, including abnormal results from laboratory analyses, that is not reasonably related to the use of the intervention/investigational medicinal product.

Those AEs which are suspected of having a causal relationship to the investigational medicinal product will be followed up until the subject has recovered or is well taken care of and on the way to good recovery (see also section 9.4, Follow-up of Adverse Events).

If the reporting investigator does not provide any information on causality, the sponsor should consult with the reporting investigator and encourage the expression of a position on this issue. The sponsor must take into account the assessment of causality provided by the investigator. If the sponsor disagrees with the investigator's assessment of causality, both the investigator's and the sponsor's views should be included in the report.

9.2.2. Assessment of intensity

Information on all adverse events should be recorded immediately in the Adverse Event module of the eCRF using the NCI.CTCAE version 5.0. All adverse events occurring during the study period must be recorded. The clinical course of each event should be followed until resolution or stabilization. Serious adverse events which are still ongoing at the end of the study period must be followed to determine the final outcome.

Any serious adverse event, which occurs after the study period and is considered

possibly related to study treatment or study participation, should be recorded and reported immediately.

The grade as assessed by the investigator according to the definitions in NCI CTCAE version 5.0:

Grade 1 = mild: Asymptomatic or mild symptoms. Clinical or diagnostic observations only. Intervention not indicated.

Grade 2 = moderate: Minimal, local or noninvasive intervention indicated. Limiting age-appropriate instrumental ADL (preparation of meals, shopping for groceries or clothes, using the telephone, managing money, etc).

Grade 3 = severe: Severe or medically significant but not immediately life-threatening. Hospitalization or prolongation of hospitalization indicated. Disabling. Limiting self care ADL (bathing, dressing and undressing, feeding self, using the toilet, taking medications, and not bedridden).

Grade 4 = life-threatening or disabling

Grade 5 = death related to AE

9.2.3. Assessment of seriousness

The investigator is responsible for assessing the seriousness (serious or non-serious). If the adverse event is considered serious, this should be reported as a serious adverse event (SAE) by the investigator to the sponsor.

9.3. Reporting and registration of Adverse Events

At each study visit, adverse events (AE) are registered, starting at study enrollment, up to 1 year after last treatment (ICI, surgery or BRAF/MEK). All AE that occurs during the study and which are observed by the investigator/study nurse or reported by the subject will be recorded in the medical record and in the CRF regardless of whether they are related to the intervention or not. Assessment of causal relationship, severity, and whether the AE is considered an SAE or not will be made by the investigator. At minimum, for each AE/SAE, a description of the event is recorded (diagnosis/symptom if diagnosis is missing), start and stop dates, causal relationship, severity, if the AE is considered an SAE or not, measures and outcome.

9.3.1. Reporting of Adverse Events (AE)

AEs will be recorded on a separate AE form in the CRF, with complete information regarding seriousness, intensity and causality and all AE assessments will be made by an investigator. Expected adverse events based on knowledge of the disease in question and expected clinical course are not to be reported as AE in this clinical study.

The following events should NOT be recorded as AEs:

• Symptoms, findings, events, conditions or medical interventions that are deemed to be related to disseminated melanoma with NO relation to the study treatments.
• Symptoms, findings, events, conditions or medical interventions that are deemed to be related to other previously known illness with NO relation the study treatments.
• Laboratory findings outside reference intervals that are deemed to be off no clinical significance.

9.3.2. Reporting of Serious Adverse Events (SAE)

SAEs wills be reported by the investigator to the sponsor on a separate SAE form within 24 hours after the SAE has been communicated to the investigator. Follow-up information describing the outcome of the SAE and actions taken will be reported as soon as available. Assessment of the SAE being expected for the intervention or not will be made by the sponsor in agreement with the principal investigator. The original SAE form must be filed in the investigator site file.

Events that are commonly related to the malignant disease are exempted from SAE reporting. For this reason, the following events should not be reported:

• Death, if due to progression of the cancer
• Progression of disease
• Inpatient hospitalization if due to expected cancer morbidity as judged by the investigator

The following minimum information is required for any reported SAE:
• Subject number, sex and age
• The date of SAE
• A description of the SAE (event, seriousness of the event)
• Causal relationship to the treatment
• Identifiable details of reporter/Investigator

9.3.3. Reporting of Suspected Unexpected Serious Adverse Reactions (SUSAR)

The Sponsor must evaluate each SAE and decide whether the event is a SUSAR or not. An SAE assessed by sponsor to be a SUSAR must be reported to the European Medicines Agency (EudraVigilance database) according to the specified time frames. The SUSAR reporting will be done by sending the CIOMS form to the Swedish Medical Products Agency.

SUSARs that are fatal or life-threatening are reported as soon as possible and no later than 7 days after the incident has become known to the sponsor. Relevant follow-up information is sent thereafter within an additional 8 days. Other SUSARs are reported as soon as possible and no later than 15 days after they have come to the sponsor’s knowledge.

9.4. Follow-up of Adverse Events

Reportable AE and SAE are followed up until they are fully evaluated or no longer considered clinically non-significant by the principal investigator.

All reported adverse events (AE/SAE) that have not been resolved by the end of the trial are to be followed until the event is resolved/stable/persistent. The follow-up information should describe whether the event has resolved or continues, if and how it was treated, and whether the patient continued or withdrew from trial participation. If an AE/SAE continues after end of trial, a final assessment will be done by the principal investigator and the patient will be followed until the symptoms disappear or they reach a stable state.

Laboratory toxicities (e.g., suspected drug induced liver enzyme evaluations) will be monitored during the follow-up phase via on site/local labs until all study treatment-related toxicities resolve, return to baseline, or are deemed irreversible.

9.5. Independent Data Monitoring Committee

The steering committee will appoint a data monitoring committee (DMC), consisting of three independent clinicians with experience in the management of patients with melanoma metastases and monitoring of randomized clinical trials.

The DMC will ensure the safety as well as the general execution of the trial on behalf of the trial participants. The drugs under investigation have market approval by the authorities and the safety profile of the treatments are well

established. Therefore, we consider the risk of unexpected side effects or adverse events related to the treatment to be low.

Outcome analysis may be performed on request of the DMC. There will be no formal rules for discontinuation of enrolment based on statistical considerations. A recommendation to discontinue enrolment into the trial should reflect a strong conviction of the DMC that the risk to the current and future trial patients not yet enrolled outweighs the potential impact of premature termination on future clinical practice. If the DMC recommends early termination of the trial, they will inform the coordinating PI and Sponsor through written documentation of such a decision and the rationale within five working days after the meeting.

The responsibilities of the DMC will be detailed in a separate charter agreed upon by the steering committee and the DMC members.

9.6. Annual Safety Report (ASR)

The sponsor will submit a single annual safety report for all investigational medicinal products used in this trial via CTIS. Since the sponsor is non-commercial, the marketing authorization holder of the investigational product is within EU/EEA and the SPC is used as RSI, the Simplified template of Annual Safety Report will be used. The annual report will contain aggregated and anonymized data. A summary assessment of the safety situation for the subjects and a benefit/risk evaluation for the trial will be included. The ASR will be accompanied by the RSI in force at the start date of the report. If significant changes in the RSI have occurred during the reporting period, these will be listed in the ASR.

9.7. Procedures in case of emergencies, overdose or pregnancy

Medication errors, pregnancy and uses other than those specified in the CTP, including misuse and abuse of the investigational medicinal product, shall be subject to the same reporting obligations as adverse reactions.

If an unforeseen event is likely to have a serious impact on the benefit/risk relationship, the sponsor and investigator should take appropriate Urgent Safety Measures (USM) necessary to protect the subjects. Examples of such measures are to temporarily suspend the clinical trial or to introduce supplementary monitoring measures. The sponsor should notify Medical Product Agency, and

via CTIS, inform the concerned Member States about the event and the measures taken. Notification must be made as soon as possible, but no later than seven days after the measures have been taken.

If, following initiation of study treatment in this trial, a subject who participates in this clinical trial becomes pregnant, or may have been pregnant at the time of study treatment exposure, including during at least 5 half-lives after product administration, this person must be followed up until the birth has taken place. If the fetus/child has any congenital malformation, this must be reported as a serious adverse event (SAE).

The investigator must immediately notify the sponsor of this event. If the investigator determines a possible favorable benefit/risk ratio that warrants continuation of study treatment, or re-initiation of study treatment, a discussion between the sponsor and the investigator must occur. If, for whatever reason, the pregnancy has ended, confirmed by negative serum pregnancy test, treatment may be resumed (at least 3 weeks and not greater than 6 weeks after the pregnancy has ended), following approvals of participant/sponsor/IRB/EC, as applicable.

Follow-up information regarding the course of the pregnancy, including perinatal and neonatal outcome and, where applicable, offspring information must be reported.

Any pregnancy that occurs in a female partner of a male study participant should be reported to Sponsor or designee.

In cases where a study drug can be present in seminal fluid, at exposures sufficient to potentially cause fetal toxicity, and if any sexual activity (e.g., vaginal, anal, oral) has occurred between a male participant and a pregnant WOCBP partner(s), the information should be reported to the Sponsor or designee, even if the male participant has undergone a successful vasectomy.

10. Statistics

The sections below summarize the intended statistical methods and analyses for this study. A more detailed Statistical Analysis Plan (SAP) will be written and finalized prior to any lock of the study database and any analysis performed. The SAP will give a detailed description of the summaries and analyses that will be performed and will clearly describe when these analyses will take place.

.

10.1. Analysis population

Two populations will be defined for data analysis: the intention to treat (ITT) population, and the per-protocol (PP) population. The ITT population comprises all randomized patients, and the PP population includes only those subjects who completed the intervention originally allocated. All the analysis (primary and secondary endpoints) will be performed on both the ITT and the PP population.

10.2. Statistical analyses

10.2.1. Statistical methods

All measurements will be analyzed based upon the type of distribution, and descriptive statistics will be presented by assessment time point, as appropriate. Descriptive statistics for continuous variables (number [N], mean, median, standard deviation [SD], quartiles, minimum, and maximum), descriptive statistics for categorical variables (N and percentage), and individual subject profiles will be presented, as appropriate. Missing values for safety and exploratory outcomes will be treated as missing, unless stated otherwise. For the primary endpoint EFS, and the secondary endpoints RFS, DMFS and OS the time to event approach will be used in the Kaplan-Meier approach with the corresponding log-rank test and stratifying for transit metastasis. In addition, Cox regression models will be estimated for each time to event endpoint with the calculation of Hazard Ratios (HR) and the corresponding 95% confidence intervals (95% CI) between the two arms. A statistical significance will be considered at a two-sided 5% significance level.

10.2.2. Drop-outs

Since the impact of missing data is expected to be small, no multiple imputation method for missing data is planned. However, the issue of missing data could arise; the choice of the imputation method for missing data will depend on the pattern of missing data and the type of the imputed variable. This will be specified in the SAP.

10.3. Adjustment of significance and confidence interval

The study has only one primary endpoint, and no analysis will be corrected for multiple comparisons.

10.4. Sample size calculations

The study design is a superiority trial comparing anti-PD-1 monotherapy versus anti-CTLA-4/anti-PD-1 blockade in resectable stage III-IV melanoma with event-free survival (EFS) as the primary endpoint. For the sample calculation we have assumed the following:

- Relapse free survival at two years
  - 62% for monotherapy (corresponds to an underlying hazard rate of 0.14)
  - 75% for anti-CTLA-4/anti-PD-1 blockade (corresponds to an underlying hazard rate of 0.24)
- Accrual time: two years
- Follow up time: five year of follow up
- Power: 70%
- Type 1 error: 5%

Based on these assumptions the total sample size will be 128 subjects (64 per arm), with an expected total of 85 events.

10.5. Interim analysis (if relevant)

Not relevant since there will not be an interim analysis.

11. Quality Control and Quality Assurance

The clinical trial will be conducted in accordance with the CTR/clinical study protocol, the ethical principles of the Declaration of Helsinki and current national and international regulations. This is to ensure the safety and integrity of the subjects as well as the quality of the data collected. The sponsor is maintaining quality assurance to ensure that the study is conducted, and data are generated, documented (record), and reported in compliance with the protocol, GCP, and applicable regulatory requirement(s).

11.1. Quality Assurance and Sponsor oversight

The Sponsor will have quality assurance systems with communication plan for all involved, training of research nurse as well as physicians, we will develop working manuals, starting and ending meetings. Moreover, both monitoring and audit for some of the crucial variables will be performed according to a monitoring plan to have the best trial's quality. The trial will be monitored by an independent monitor and the monitor will be appointed by the Sponsor as per agreed in the monitoring plan. For detailed monitoring, see section 11.2.

The investigators will allow trial-related monitoring, auditing, and regulatory inspections by providing access to the CRF, subject's medical record and other source data and other trial specific documentation. See also the Subject Information and Informed Consent Form.

The Principal Investigator will ensure that all aspects of the protocol are followed, including the randomisation procedure, the accurate recording of results, the reporting of Adverse Events, Product Accountability and record keeping.

11.2. Monitoring

The clinical trial will be monitored by an independent monitor before the trial begins, during the trial, and after the trial has been completed. This is to ensure that the trial is carried out according to the protocol and that data is collected, documented, and reported according to ICH-GCP and applicable ethical and regulatory requirements. Monitoring is performed as per the trial's monitoring plan and is intended to ensure that the subject's rights, safety, and well-being are met and that data in the CRF are complete, correct, and consistent with the source data.

The monitor will have regular contacts with the sites to verify that: informed consent has been signed prior to execution of any trial-specific actions, that subjects are included according to the protocol's inclusion and exclusion criteria, that the trial's main parameters and safety reporting are handled correctly and to verify that the trial's essential documents are complete (according to chapter 8, ICH-GCP). Queries will be issued electronically.

The investigator should ensure that all persons assisting with the trial are adequately informed and trained in the protocol, the investigational products and their trial related duties and factions. The investigator will ensure the collection of CVs for all personnel involved in this trial. All site personnel participating in trial-specific tasks will undergo ICH-GCP training, and no study-related activities will

be conducted until delegation has been granted. This information will be recorded on the delegation log and kept in the ISF. Investigators and other responsible personnel must be available during the monitoring visits, audits and inspections and should devote sufficient time to these processes. The monitor will verify that training has been performed and that this is documented. The monitor will also ensure source data verification (comparison of the data in the eCRF with the medical records and other source data). The monitor must have direct access to source data. The confidentiality of the subjects' identities shall be well protected consistent with local and national regulations when the source documents are subject to direct access. The extent of monitoring will be defined in a monitoring plan based on the identified risks of trial.

11.3. Source data

The investigator must keep source documents for each subject in the trial. A document describing what has been classified as source data for each variable in the trial (source data reference document) will be included in the Investigator Site File (ISF). The investigator must ensure that all source documents are accessible for monitoring and other quality control activities.

Source data is defined before trial start at each individual site and can, in cases where source data is not registered in another document, consist of the CRF. This should be decided in consultation with the monitor and clearly stated in the source data reference document.

Access to trial-related documentation, such as subjects' medical records, CRFs, other source data and other trial documentation will be provided for monitoring and auditing purposes. Access to subjects' medical records will require a secrecy agreement to be signed by the person responsible for the medical records at the trial site and by the monitor and auditor, if applicable. Access will also be granted in the context of regulatory inspections. Subjects must provide informed consent for monitoring, auditing and other regulatory inspections by signing the Subject Information and Informed Consent Form where this is specified.

11.4. Deviations, serious breaches and other reporting obligations

Investigator(s) are not allowed to deviate from the study protocol except if it is for the protection of the subjects' rights, safety, or well-being under emergency

circumstances. The responsible investigator shall, without delay, report to the sponsor any serious breaches and deviations from the trial protocol, ICH-GCP and other regulations that significantly and directly affect, or with high likelihood could affect, the subjects' safety and integrity or the reliability and robustness of the data generated in the trial. The sponsor should assess the suspected serious breach and the consequences of deviations that have occurred, and, without undue delay but no later than 7 days (from knowledge) report to the appropriate regulatory authorities.

Other unexpected events that may affect the benefit/risk relationship must be reported to the appropriate regulatory authorities without undue delay, but no later than 15 days after the sponsor becomes aware of the event.

Minor deviations that do not affect subjects' integrity or safety, nor significantly affect the trial's scientific value, are documented in the trial documentation of the principal investigator and the sponsor and appropriate measures shall be taken. Major deviations must be recorded in the clinical trial report.

11.5. Audits and inspections

Authorized representatives for the sponsor and Competent Authority (CA) may carry out audits or inspections at the trial site, including source data verification. The investigator must ensure that all source documents are available for audits and inspections. The purpose of an audit or inspection is to systematically and independently review all trial-related activities and documents, to determine whether these activities were performed, registered, analyzed and reported correctly according to protocol, ICH-GCP and applicable regulations. The auditor(s), and the CA must be granted access to the subject's original medical records for verification of clinical trial procedures and/or data, without violating the confidentiality of the subject, to the extent permitted by the applicable laws and regulations.

12. Ethics

12.1. Compliance to the protocol, ICH-GCP and regulations

The trial will be performed in compliance with this clinical trial protocol, the EU regulation on clinical trials on medicinal products for human use (536/2014), the Declaration of Helsinki, ICH-GCP (Good Clinical Practice), and current national

regulations governing this clinical trial. This is to ensure the safety and integrity of the trial subjects as well as the quality of the data collected.

12.2. Ethical review of the trial

The final protocol and the Subject Information Sheet and Informed Consent Form must be approved, as a part of the application for a permit for clinical trials for medicinal products via CTIS. Approval will be obtained from the Ethical Review Authority and the Medical Products Agency before the trial can be conducted. The authority must be informed of any changes to the trial protocol in accordance with current requirements.

12.3. Procedure for obtaining informed consent

The principal investigator at each site shall ensure that the subject is given full and adequate oral and written information about the trial, its purpose, any risks and benefits as well as inclusion and exclusion criteria. Subjects must also be informed that they are free to discontinue their participation in the trial at any time without having to provide a reason. Subjects should be given the opportunity to ask questions and be allowed time to consider the provided information. If the person chooses to participate, both the subject and the investigator shall sign the informed consent form. The investigator must be a delegated physician. A copy of the subject information as well as the informed consent form shall be provided to the subject. The subject’s signed and dated informed consent must be obtained before any trial-specific activity is performed. Documentation that the informed consent was signed and dated prior to study inclusion must be entered into the medical records at the time the informed consent is obtained. The original, signed Informed Consent Form (ICF) must be stored in the Investigator Site File.

Each subject who participates in the trial will be identified by a subject number on a subject identification list. The subject agrees that monitors, auditors, and inspectors may have access to their medical records and other source data. If new information is added to the trial, the subject has the right to reconsider whether he/she will continue their participation. This occurs by allowing the subject to sign a revised subject information and informed consent form.

12.4. Data protection

The personal data controller is obliged to take measures to ensure that the General Data Protection Regulation (GDPR) is followed, to describe built-in data protection features and security when processing and to report personal data breaches as per legislation to the competent authority.

Appropriate technical and organizational measures shall be taken to protect the personal data and processed information from unauthorized access, disclosure, dissemination, alteration or destruction and from accidental loss, in particular where the processing involves the transmission of data over a network.

If any part of the data is processed by another organization, inside or outside the European Union, appropriate agreements and/or other appropriate protective measures are taken to ensure that the data processing is performed in accordance with the provisions of the General Data Protection Regulation (EU ordinance 2016/679, GDPR) and other relevant legislation, before any data transfer takes place.

In the information provided to subjects, subjects will be fully informed about how their trial data will be collected, used and disclosed. The content of the informed consent form complies with relevant integrity and data protection legislation. The subject information and the informed consent form will explain how trial data are stored to maintain confidentiality in accordance with national data legislation. The informed consent form will also explain that for verification of the data, representatives delegated by the sponsor, as well as relevant authorities, may require access to parts of medical records or trial records that are relevant to the trial, including the subject’s medical history.

Each study center will enter all study data into an eCRF. The sponsor will be responsible for all data registrations, statistical programming, and analysis as well as statistical quality control and validation of programming and statistical analysis. The sponsor will be responsible for the collected data in the study.

All patient data collected and processed for the purposes of this study will be managed by the sponsor with adequate precautions to ensure the confidentiality of those data, and in accordance with applicable national and/local laws and regulations on personal data protection. All information processed by the sponsor will be pseudonymized and identified with a specific trial code. All subjects are registered in a subject identification list (subject enrolment and identification list) that connects the subject’s name and personal number with a specific trial code. In any presentations of the results of this study at meetings or in publications, the patients’ identity will remain confidential.

12.5. Insurances

The study subjects are covered by the Patient Insurance and the Pharmaceutical Insurance.

13. Substantial changes to the trial

Substantial changes to the clinical trial protocol may not be implemented before authorization has been granted by the relevant authority via CTIS. It is the responsibility of the sponsor to assess whether a change is substantial or not.

The investigator must not make any deviation from or change of the protocol, except when it is necessary to eliminate an immediate risk to the trial subjects, or where the changes only include logistical or administrative aspects of the trial. Other deviations/changes besides the abovementioned required agreement with the sponsor and documented authoritative opinion regarding the amendment from relevant authorities.

In the event that substantial changes to the protocol which may affect the safety, rights of subjects or the reliability and robustness of data generated need to be implemented during the course of the trial, authorisation from the relevant authorities of a substantial modification in CTIS must be granted. A substantial change includes e.g. the addition of a new trial site or a change of the principal investigator at the trial site.

Non-substantial modifications are submitted in CTIS in the next substantial modification application.

14. Collection, handling, and archiving of data

Data is collected in the eCRF. The investigator is responsible for data accuracy and completeness. The study monitor will compare source data to the eCRF. Queries will be created electronically by the monitor.

The clinical trial master file shall at all times contain the essential documents relating to the clinical trial in order to allow verification of the conduct of the clinical trial and the quality of the data generated. It shall be readily available, and directly accessible upon request, to the Member State Competent Authority.

The sponsor is responsible for keeping the documentation for the entire trial. The principal investigator shall keep an Investigator Site File with all trial documentation for the site. The files should have relevant content according to the trial and follow ICH-GCP chapter 8 “Essential documents”. The principal

investigator will store the trial site's data, subject identification list, original of the subject information sheet and obtained trial consent inaccessible to unauthorized persons, but such that trial subjects can be identified by those responsible for the trial. This information must not be stored at the sponsor.

Subjects who participate in the trial are coded with a trial-specific identification number. All subjects are registered on a subject identification list (subject enrolment and identification list) that connects the subject's name and personal number with a subject number/trial identification number.

All data will be registered, managed, and stored in a manner that enables correct reporting, interpretation, and verification. The complete Trial Master File with essential documents will be archived for at least 25 years according. Source data in the medical records system are stored and archived in accordance with national regulations.

The investigator agrees to obtain the sponsor's agreement prior to disposal, moving, or transferring of any study-related records.

14.1. Case Report Form

An electronic Case Report Form (eCRF) is used for data collection. The eCRF system REDCap is used in this trial. All data saved in the REDCap system will be stored at VGRIT-servers. The investigator must ensure that data is registered and any corrections in the eCRF are made as stated in the clinical trial protocol and in accordance with the instructions. The investigator must ensure that the registered data is correct, complete, and that reporting takes place according to the timelines that have been predefined and agreed. The principal investigator at each site signs the completed eCRF. A copy of the completed eCRF will be archived at the site.

15. Notification of trial completion, reporting, and publication

End of the trial is reported in CTIS at the latest 15 days after completion.

Within one year of trial completion, a clinical study report is completed, and a summary of the clinical trial results must be reported in CTIS, including a summary for laypersons.

The clinical trial report with individual data will be completed by the sponsor and provided to Principal investigators. The clinical trial report shall be archived in the

Trial Master File by the sponsor and by the principal investigator at each site, in their Investigator Site Files, throughout the entire retention period, and available on request for inspections by the authorities. The clinical trial report must contain sufficient information so that the Medical Products Agency or other authorities can make a complete evaluation of the trial conduct and the results.

A published article is not to be equated with the summary report to CTIS or the full clinical trial report.

If the clinical trial is interrupted or terminated prematurely the sponsor will report to the Medical Products Agency within 15 days together with a justification. If the sponsor has temporarily halted or prematurely terminated the clinical trial on safety grounds, the Medical Products Agency will be informed within 24 hours.

The final clinical trial report shall include detail with respect to the temporary halt. The reasons for such action and follow-up measures must be provided. The resumption of a clinical trial after its temporary interruption due to a change in the benefit/risk balance is considered a substantial modification.

Interruptions that do not affect the benefit/risk relationship must be notified, via CTIS, within 15 days, stating the reason for the interruption. Notification of restart shall be made, via CTIS, within 15 days. See also section, 5.4.1. Premature termination of the clinical trial and 6.4 Withdrawal criteria.

The results of the study will be published on ClinicalTrials.gov, in peer-reviewed scientific journals and be presented at scientific symposia and congresses

16. References

1. Arnold M, Singh D, Laversanne M, Vignat J, Vaccarella S, Meheus F, et al. Global Burden of Cutaneous Melanoma in 2020 and Projections to 2040. JAMA Dermatol. 2022 May 1;158(5):495–503.

2. Eggermont AMM, Kicinski M, Blank CU, Mandala M, Long GV, Atkinson V, et al. Five-Year Analysis of Adjuvant Pembrolizumab or Placebo in Stage III Melanoma. NEJM Evid. 2022 Nov;1(11):EVIDoa2200214.

3. Liu J, Blake SJ, Yong MCR, Harjunpää H, Ngiow SF, Takeda K, et al. Improved Efficacy of Neoadjuvant Compared to Adjuvant Immunotherapy to Eradicate Metastatic Disease. Cancer Discov. 2016 Dec;6(12):1382–99.

4. Amaria RN, Reddy SM, Tawbi HA, Davies MA, Ross MI, Glitza IC, et al. Neoadjuvant immune checkpoint blockade in high-risk resectable melanoma. Nat Med. 2018 Nov;24(11):1649–54.

5. Rozeman EA, Menzies AM, van Akkooi ACJ, Adhikari C, Bierman C, van de Wiel BA, et al. Identification of the optimal combination dosing schedule of neoadjuvant ipilimumab plus nivolumab in macroscopic stage III melanoma (OpACIN-neo): a multicentre, phase 2, randomised, controlled trial. Lancet Oncol. 2019 Jul;20(7):948–60.

6. Reijers ILM, Menzies AM, van Akkooi ACJ, Versluis JM, van den Heuvel NMJ, Saw RPM, et al. Personalized response-directed surgery and adjuvant therapy after neoadjuvant ipilimumab and nivolumab in high-risk stage III melanoma: the PRADO trial. Nat Med. 2022 Jun;28(6):1178–88.

7. Patel SP, Othus M, Chen Y, Wright GP, Yost KJ, Hyngstrom JR, et al. Neoadjuvant-Adjuvant or Adjuvant-Only Pembrolizumab in Advanced Melanoma. N Engl J Med. 2023 Mar 2;388(9):813–23.

8. Blank CU, Lucas MW, Scolyer RA, van de Wiel BA, Menzies AM, Lopez-Yurda M, et al. Neoadjuvant Nivolumab and Ipilimumab in Resectable Stage III Melanoma. N Engl J Med. 2024 Jun 2;

9. Lucas MW, Menzies AM, Lopez-Yurda M, Scolyer RA, van de Wiel B, Saw RPM, et al. LBA42 Distant metastasis-free survival of neoadjuvant nivolumab plus ipilimumab versus adjuvant nivolumab in resectable, macroscopic stage III melanoma: The NADINA trial. Annals of Oncology. 2024 Sep 1;35:S1233–4.
